# Upregulation of Na/H Exchanger in Astrogliosis and Early Alzheimer’s Disease Pathogenesis

**DOI:** 10.14336/AD.2024.1294

**Published:** 2025-12-15

**Authors:** Jenelle M. Collier, Shamseldin Metwally, Mary McFarland, Sanjana Krishna, Pallavi Kurella, Victoria Fiesler, Mark Stauffer, Gulnaz Begum, Julia Kofler, Dandan Sun

**Affiliations:** ^1^Center for Neuroscience at the University of Pittsburgh, School of Medicine, Pittsburgh, PA 15240, USA.; ^2^Department of Neurology, the Pittsburgh Institute for Neurodegenerative Diseases, University of Pittsburgh, Pittsburgh, PA 15240, USA.; ^3^Department of Biological Sciences, Carnegie Mellon University, Pittsburgh, PA 15213, USA.; ^4^Department of Neuropathology, University of Pittsburgh, PA 15260, USA.; ^5^Veteran’s Affairs Research Center, Pittsburgh, PA 15240, USA

**Keywords:** Amyloid-beta (Aβ), hyperactive locomotor activity, NHE1, pH homeostasis, reactive astrocytes

## Abstract

Reactive astrogliosis has been indicated as one of the earliest pathological biomarkers in Alzheimer’s Disease (AD) pathology. We previously reported that upregulation of the Na^+^/H^+^ exchanger isoform 1 (NHE1) protein in reactive astrocytes contributes to neuroinflammation and cognitive function deficits in murine models of ischemic stroke and vascular stenosis. In this study, we utilized AD human post-mortem and APP/PS1dE9 (APP) transgenic mouse brain tissues to determine whether NHE1 upregulation in astrocytes is associated with AD pathogenesis. In both AD human and APP mouse brain tissues, a significant elevation of NHE1 protein expression was detected in glial fibrillary acidic protein expressing (GFAP^+^) reactive astrocytes in cortical and hippocampal regions, compared to control groups. Furthermore, increased astrocytic NHE1 protein and GFAP protein were detected in proximity to amyloid-beta (Aβ) plaques in APP mouse brains. We then tested the efficacy of pharmacological NHE1 inhibition using its inhibitor, HOE642, in attenuating pathogenesis in APP mice. Vehicle-treated APP mice (APP.Veh) exhibited hyperactive locomotor behavior at 4-months and 7-months of age, compared to wild-type littermates (WT.Veh). In contrast, APP mice-treated with HOE642 (APP.HOE) displayed significantly lower hyperactive locomotor behavior (p<0.01). Additionally, APP.HOE mice showed decreased density of amyloid fibrils. In summary, we detected NHE1 protein upregulation in reactive astrocytes in both AD human and APP brains. Pharmacological inhibition of NHE1 protein attenuated pathological Aβ plaque density, and hyperactive locomotor behaviors in APP mice, highlighting NHE1 as a possible therapeutic target for AD.

## INTRODUCTION

Astrocytes have diverse functions to maintain brain homeostasis by supporting ionic balance and neurotransmitter reuptake, synaptic transmission, and neurovasculature unit regulation [1-3]. Recent studies suggested a direct role of astrocyte dysfunction in contributing to AD pathogenesis [4-6]. First, among detection of AD hallmarks with fluid biomarkers, brain imaging, proteomics, and metabolomics approaches [7-9], elevation of reactive astrocyte marker glial fibrillary acidic protein (GFAP) in plasma and cerebrospinal fluid (CSF) has become one of the earliest and most consistent biomarkers for AD diagnosis in conjunction with elevated phosphorylated-Tau. Reactive astrocytes are often observed in the prodromic phase of AD prior to detection of Aβ plaque overload and synaptic loss [10-12]. Secondly, in both acute and chronic neurological diseases such as ischemic stroke and AD, reactive astrocytes undergo astrogliosis transformative process with morphological, transcriptional, and functional changes, which often lead to astrocytic dysfunction [13]. Astrogliosis and astrocyte dysfunction result in glutamate reuptake dysfunction, neuronal excitotoxicity, oxidative/excitotoxic stress, loss of the blood-brain barrier (BBB) integrity, impaired calcium signaling and glymphatic system dysfunction, causing accumulation of soluble aggregates of Aβ [13-15]. However, the underlying mechanisms for reactive astrocyte dysfunction and astrogliosis-mediated contributions to AD pathological changes remain incompletely understood.

Our previous studies reported that pathological stimulation of NHE1 plays a role in astrocyte dysfunction in murine models of ischemic stroke and vascular stenosis [16, 17]. NHE1 protein mediates H^+^ efflux in exchange for Na^+^ influx, regulating astrocytic intracellular pH (pH_i_) homeostasis [16]. Because NHE1-mediated H^+^ efflux activity influences the concentration of available H^+^ in the cytosol, its activity indirectly influences lysosomal acidification and degradation of misfolded and aggregated protein fragments like Aß [18, 19]. In addition, stimulation of NHE1 activity-mediated H^+^ efflux also supports NADPH oxidase (NOX) signaling in production of reactive oxygen species (ROS) and cytokines [20]. The objective of this study is to determine whether upregulation of the NHE1 protein plays a role in AD brain astrogliosis and accumulation of aggregated Aß.

In this study, changes of NHE1 protein expression in various regions of post-mortem AD human brain tissues and transgenic APP mouse brains were characterized. Human post-mortem AD brain tissues displayed a significant increase in NHE1 protein in GFAP^+^ reactive astrocytes compared to control subjects. In APP mouse cortical and hippocampal brain regions, significantly increased expression of NHE1 protein was also found in GFAP^+^ reactive astrocytes in close proximity to Aβ plaques. In efforts to test the impact of NHE1 upregulation in AD gliosis, Aβ, and cognitive pathologies, the well-characterized NHE1 inhibitor Cariporide (HOE642) was administered to APP mice at 4 months of age. Positive effects on attenuating hyperactive locomotor function and Aβ density accumulation in hippocampal and cortical brain regions were observed by 7-8 months of age. Our results provide evidence of NHE1 protein upregulation in reactive astrocytes as well as the potential for NHE1 inhibition in attenuating astrogliosis and AD pathologies.

## MATERIALS AND METHODS

### Materials

Anti-Glial Fibrillary Acidic Protein (GFAP) antibody (Z0334, rabbit polyclonal) was from Agilent DAKO (Santa Clara, CA), anti-GFAP (GFAP) antibody (ab4674, chicken polyclonal) from Abcam Ltd (Cambridge, MA), anti-Sodium/Hydrogen Exchanger 1 *(*NHE-1) antibody (sc-136239, mouse monoclonal) from Santa Cruz biotechnology (Santa Cruz, CA). Anti-APP/β-Amyloid (NAB228) (#2S450, mouse monoclonal) was from Cell Signaling Technology (Danvers, MA). Thioflavine S (T1892-25G, practical grade) was from Sigma-Aldrich and amyloid Fibrils (OC) antibody (200-401-E87, rabbit polyclonal) was from Rockland (Limerick, PA), DAPI (4,6-diamino-2-phenylindole, dihydrochloride) was from Life Technologies Corporation (Carlsbad, CA) and NucRed™ Dead 647 ReadyProbes™ Reagent stain (TO-PRO-3 iodide, R37113) was from Invitrogen, ThermoFisher. Alexa Fluor® 488 AffiniPure Fab Fragment Donkey Anti-Mouse IgG (H+L) (715-547-003) was from Jackson Immuno Research Laboratories Inc (West Grove, PA) to reduce species reactivity in mouse tissues. Goat anti-mouse AlexaFluor 488 (A11029) and goat anti-rabbit AlexaFluor 546 (A11035) were from Life Technologies (Grand Island, NY). H&E Staining Kit (Hematoxylin and Eosin) (ab245880) and Antigen Retrieval Buffer (100X EDTA Buffer, pH 8.0, ab93680) were from Abcam (Cambridge, MA). Cariporide (HOE642) (SML 1360) and DMSO (D2650) from Sigma Aldrich (see Supplementary Table 1).

### Post-mortem Human Brain Tissue

Pre-mounted paraffin embedded human post-mortem tissue slides (60 brain samples) were provided by the Alzheimer’s Disease Research Center (ADRC) brain bank at the University of Pittsburgh and the clinical information of the subjects were included in Table 1. Amygdala (O), hippocampal (E), and neocortical (L) regions from 10 AD cases (Braak stages V-VI) and 10 control (CTRL) cases (Braak stage I-III) were used for H&E, histochemistry and immunofluorescence staining assays as well as IMARIS 3D reconstruction analysis. The usage of post-mortem human samples was approved by the Committee for Oversight in Research Involving Decedents at the University of Pittsburgh (Pittsburgh, PA, USA).

### H&E and Histochemistry

Paraffin embedded human brain tissue slides were medium heated on a hot plate for 10 min and slides were deparaffinized (100% Xylene for 10 min x3, 100% Xylene:100% Ethanol (EtOH) for 3 min) then dehydrated in EtOH dilutions (100%, 95%, 70%, 50% EtOH for 3 min each) as described in a previous protocol [21]. Slides were washed with cold tap water and incubated in heated antigen retrieval buffer (Tris-EDTA pH 8) for 15 min at 100°C in a steamer then cooled. Slides were washed with deionized H_2_O and quenched of peroxidase with H_2_O_2_ (30%) for 15 min at room temperature (RT). Slides were washed with Tris Buffered Saline (TBS) and liquid barrier around tissue slices were drawn using ImmEdgeBlue Marker Border. After being blocked with 1X PowerBlock solution for 10 min at RT, primary antibodies in TBS++ (TBS, 3% Normal Goat Serum, and 0.3% triton X) were applied to slides and incubated at 4°C overnight. Slides were washed with TBS and incubated with Vector Lab Diluted Biotinylated Secondary solution in TBS for 30 min at RT. Vector ABC solution was applied to slides for 30 min at RT. Vector NovaRED Peroxidase Substrate solution in ddH_2_O was applied to slides and incubated for 5 min. After washed with ddH_2_O, Mayer’s Hematoxylin Stain was applied to each slide for 5 min and submerged 10 times in Acetic Acid solution to stain nuclei. Slides were then washed with ddH_2_O and dehydrated (in 95%, 100% EtOH and 100% Xylenes). Slides were air dried and Nonaqueous Vectamount was applied to specimens and covered with glass coverslip and left to dry overnight in the dark at 4°C.

**Table 1:** Human Post-Mortem Tissue Description.

Category	CW #	AGE	SEX	Braak NFT stage	Amyloid angiopathy	Arteriolosclerosis	Lewy body disease pathology	LATE pathology	Other diagnoses
AD	CW18-019	81	M	6	none	severe	Limbic	Stage 1	Lacunar infarct
AD	CW19-018	64	M	6	mild	mild	Amygdala	Stage 2	Capillary teangiectasias, pons
AD	CW19-020	81	M	6	mild	severe	Amygdala	Stage 2	Venous angioma
AD	CW20-016	78	F	6	moderate	moderate	Neocortical	Stage 1	ARTAG; multiple remote microinfarcts; meningioma
AD	CW20-021	74	F	6	mild	severe	Neocortical	Stage 2	remote cerebellar infarct
AD	CW20-132	69	F	6	moderate	mild	Amygdala	Stage 1	N/A
AD	CW21-008	81	M	5	mild	mild	Limbic	none	Remote frontal microinfarct
AD	CW21-014	97	M	5	mild	mild	none	Stage 2	ARTAG
AD	CW21-168	94	M	5	mild	severe	Neocortical	Stage 2	ARTAG
AD	CW22-123	84	M	5	mild	mild	Limbic	Stage 2	ARTAG; acute occipital microinfarct
Control	CW11-061	86	F	1	mild	moderate	none	none	N/A
Control	CW11-105	76	F	2	none	none	none	none	N/A
Control	CW12-041	83	F	2	none	mild	Brainstem	none	Acute transentorhinal microinfarct; subarachnoid hemorrhage bilateral convexities
Control	CW12-071	90	F	3	none	mild	none	none	Remote microinfarct internal capsule
Control	CW14-025	92	F	1	none	mild	none	none	N/A
Control	CW15-029	80	F	3	none	moderate	none	none	N/A
Control	CW16-019	87	M	2	mild	mild	none	none	ARTAG; acute ischemic encephalopathy; remote infarct
Control	CW16-028	90	M	1	none	mild	Brainstem	none	Intraparenchymal cerebellar hemorrhage; subarachnoid hemorrhage
Control	CW19-017	91	M	2	none	moderate	none	none	ARTAG; remote cerebellar microinfarct

### Immunofluorescence Staining of Human Brain Tissues

After deparaffinizing and blocking steps as described above, mounted brain sections were circled with a Blue Marker Border (ImmEdge Pen Vector) around the tissue sections. 400µL Power Block was applied to each slide for 10 min. Primary antibodies prepared in TBS++ (300 µL) were applied to each slide and incubated overnight in the dark at 4°C. Each slide was washed three times with TBS and incubated with fluorescent secondary antibodies in TBS++ (1:200) at RT for 45 min. Slides were washed three times with TBS and incubated in DAPI (1:1000 TBS) for 15 min at RT. After washing with TBS, TrueBlack (1:20 in 70% EtOH) was applied to each slide for 30 seconds and washed three times with TBS to decrease the autofluorescence resulting from lipofusion. Slides were placed on hot plate to allow for partial drying on medium heat before being mounted using Vectashield Mounting Medium.

### APP/PS1dE9 Colony and Brain Tissue Immunostaining

All animal studies were approved by the University of Pittsburgh Institutional Animal Care and Use Committee, which adhere to the National Institutes of Health Guide for the Care and Use of Laboratory Animals and are in accordance with the Animal Research Reporting In Vivo Experiments (ARRIVE) guidelines [22]. C57BL/6J (JAX#00064) and transgenic APP/PS1dE9 (B6.Cg-Tg(APPswe, PSEN1dE9) 85Dbo/Mmjax (#034832-JAX) mice were purchased through the Mutant Mouse Research and Resource Center (MMRRC) and bred in house at the University of Pittsburgh Division of Laboratory and Animal Resources (DLAR) animal research facility. All wild-type (WT) results in our data were collected from WT littermates of APP mice. Genotyping was performed following the standard PCR reaction protocol provided by the Jackson Laboratory. Briefly, primers for the WT forward (42431; 5’- GTG TGA TCC ATT CCA TCA GC -3’), Common (42432; 5’- GGA TCT CTG AGG GGT CCA GT -3’), and mutant forward (42433; 5’- ATG GTA GAG TAA GCG AGA ACA CG -3’) were used. The expected mutant allele base pair (bp) size is 142 bp and the expected WT allele size is 256 bp. APP mice were bred to be heterozygous. The colony mortality rate in APP mice was ~30%, consistent with other reports [23, 24]. At 4, 6, 8, or 10 months old, WT and APP mice were euthanized by CO_2_ and perfused with Dulbecco’s phosphate buffered saline (PBS) followed by cold 4% paraformaldehyde (PFA). Mouse brain tissue was harvested and transferred to a 30% sucrose solution where they were stored at 4°C overnight. Coronal brain sections (25µm thickness) were obtained by a Physitemp temperature regulated Leica SM2010R Microtome. Brain sections (at the level of the hippocampus and cortex, distance from bregma -2.18 mm +/- 0.24 mm) were washed 3 times for 5 min each with TBS and then incubated in TBS++ blocking buffer (300µL) for 1 hr. The procedures for primary and secondary antibody incubation were performed as described above.

### Confocal Image Acquisition and Analysis

Immunofluorescence Z-stack images were captured using the NIKON A1R confocal microscope with the 40x oil-immersion objective at 1024 × 1024-pixel resolution (0.103 μm/pixel). Cell analysis and quantification were conducted using FIJI. Before quantification was assessed, all the channels collected within an image were corrected for background noise using background subtraction. Minimum and maximum image intensities were adjusted to produce a clear image of all channels used in immunofluorescence staining. Using the cell-counter plug-in, the total number of nuclei (DAPI, or TO-PRO 3 dyes), the total number of reactive astrocytes (GFAP^+^ in TRITC (546) channel), or the total number of reactive astrocytes exhibiting NHE1 upregulation/increased FITC (488) channel intensity was quantified in human and mouse brain tissue sections. All negative control images with secondary antibody staining were captured on the NIKON AIR confocal microscope and described in Supplementary Figure 3.

### Imaris Software Analysis of 3D Cell Reconstruction

Z-stack analysis and 3D cell reconstruction were executed using Imaris 10.0.1 program. Channel 3 - TRITC was selected as the source channel to create 3D structures from the astrocyte GFAP staining channel. After adding a new filament project, the “autopath (no loops) & no spines” option was selected. Region of interest (ROI) was created per image file to select astrocytes for 3D reconstruction and analysis. The diameter of the soma, within the selected ROI was measured and used as the value for the “estimated largest diameter”. The starting points threshold was adjusted to include only one seed point per soma. The “Calculate Soma Model” was selected, and the soma models were used for the soma segmentation step. The “Multiscale points” option was enabled, and the thinnest diameter was set as 0.2µm and the largest as 3µm. The seed points threshold was adjusted based on each Z-stack. Once 3D astrocytes were reconstructed, soma volume, total process volume, and number of process branch points were quantified.

### Thioflavine S Staining and Analysis of Astrogliosis Surrounding of Aβ Aggregates

After incubation with Topo 3 in TBS (1:1000) for 15 min and washing with TBS and ddH_2_O, 0.05% Thioflavine S solution (sterile filtered through 0.2µm syringe filter) was added to free floating brain slices and incubated for 20 min in the dark at RT. After washing in 70% ethanol three times for 5 min, slides were washed in TBS and placed on hot plate to allow for partial drying before being mounted using Vectashield Mounting Medium. Thioflavine S-stained APP mouse brain slices were imaged on Nikon confocal as previously described. To quantify astrocytic NHE1 expression in relation to Aβ plaques, a circular ROI (a radius of 15µm from the center of a Thioflavine S labeled Aβ plaque was created using ImageJ software to measure mean intensity of GFAP and NHE1 in astrocytes surrounding Aβ plaques (Aβ+) in cortical and hippocampal areas of the APP mouse brains (8-10-month-old). Control ROI areas, without a presence of Aβ plaque aggregates (Aβ-) were selected for comparison. WT mouse brains did not develop Aβ plaques and were not processed. To assess the overlapping intensity profiles of NHE1 and GFAP in reactive astrocytes surrounding the Aβ plaques, Nikon Elements Analysis Software was used. A single middle slice of the z-stack image was selected, then an intensity profile polyline was drawn through the region of highest intensity for Thioflavine S to measure an Aβ (+) region [25, 26]. The line drawn through the plaque or ROI of Aβ (-) negative control was twice the diameter of the plaque in order to measure the intensity profile of overlapping NHE1 and GFAP immunoreactive signals in astrocytes.

Z-stacks analysis and 3D cell reconstruction were also executed to analyze reactive astrocyte volume in proximity to Aβ plaque aggregates using IMARIS 10.0.1 program. Using stacked images from 8 and 10-months old APP brains, an extended surface with a radius equal to the diameter of the plaque, was created using the 3D reconstructed plaque’s perimeter as a starting point. One surface of NHE1^+^ reactive astrocytes was chosen according to the immunostaining image. Another surface was created to reconstruct from the GFAP channel to measure reactive astrocyte volume. Next, a spots creation was selected to reconstruct the NHE1 signal into individual 3D spots. After a series object-to-object surface recreations between the extended plaque surface, GFAP surface, and NHE1 spots, the number of NHE1 spots within the GFAP volume that was only overlapping with the extended plaque surface was specifically selected and measured. The % of GFAP volume and % NHE1 spots within GFAP volume within the plaque extended surface was calculated for each image from the entire 3D field of view (1024x12024x25).

### NHE1 Inhibitor HOE642 Administration

WT littermates and APP mice (male/female) at 4-months of age were randomly assigned to receive either dimethyl sulfoxide vehicle (Veh) control (100% DMSO dissolved in PBS) or NHE1 inhibitor Cariporide (HOE642, 10mg/ml dissolved in DMSO). Veh (6.5% DMSO in PBS) or HOE642 (0.3 mg/kg/day) was administered via osmotic mini pump (Alzet, type 2004, Durect corporation, Cupertino, CA), which was subcutaneously implanted (at upper back below dermal surface) [17, 27, 28]. Each pump (200µL volume) supplied for Veh or HOE642 for 3 weeks and two pumps were sequentially implanted for each mouse to ensure 6 weeks of delivery. Behavioral testing was performed in mice at 4 months of age (baseline assessment prior to drug treatment) and at 7 months of age (post-treatment assessment). After completion of behavioral testing, mouse brains were harvested at 8 months of age to measure changes in astrogliosis mean intensity and Aβ plaque diameter and accumulation.

### Assessment of Mouse Behavior

Behavioral assays were conducted at the University of Pittsburgh Preclinical Phenotyping Core (PPC) [29].

#### Open Field Test

Each mouse was placed in the center of an illuminated open field chamber (50 cm × 50 cm × 50 cm) and ran for 1 hour after 1 hour habituation period in behavior testing room. Total travel distance, vertical activity, and margin time were analyzed for assessment of general locomotor activity and anxiety.

#### Light Dark

After 1 hour of habituation, each mouse was placed in the covered dark half of an open field chamber facing the door toward the illuminated open half of the open field chamber. Light and dark chambers were equally sized with an open door to move freely throughout each side. Each session was run for 10 min and duration of time spent in dark chamber and number of crossovers into the dark chamber were measured This test is used to assess anxiety-like behaviors in mutant rodent models based on the natural aversion of mice to brightly illuminated areas and on their spontaneous exploratory behavior in novel environments.

#### Light Dark

*Novel Spatial Recognition*

The Y-maze apparatus consists of three identical arms, which join in the middle to form a “Y” shape. A 10-min training period was performed where each animal started in the vertical arm (start arm) with one of the other two arms closed (novel arm). After a 10-min interval, the mice were returned to the Y-maze and placed in the same starting arm and allowed to freely explore all three arms of the maze for 5 min to assess the Novel Spatial Recognition (NSR).

### Statistical Methods

All data collection was performed blinded to experimental group assignments. Statistical analyses were performed using GraphPad Prism 9-10.1.2 (GraphPad Software, Inc., CA, USA). Each data set passed the Shapiro Wilk’s Test for Normality distribution before running further statistical analysis. Data in figure 6 were analyzed with Kolmogorov-Smirnov and in figure 7 with nonparametric Mann-Whitney T-test. All data are represented as mean and standard error of mean (SEM). A *p* < 0.05 was considered statistically significant, and adjusted p-values for each test were reported. Each data set used a 95% Confidence Interval. *N* values represent the number of individual brains used for each experiment. Multiple unpaired, parametric two-tailed T-test with Sidak-Bonferroni method was used for all AD human brain immunohistochemistry staining analyses and IMARIS 3D analyses. For three or more groups, ordinary two-way ANOVA was conducted for multiple comparisons using Bonferroni method correction for AD mouse immunohistochemistry, Aβ proximity, behavior assays, and Aβ immunostaining experiments. A total of 125 mice were used for the experiments of this study. Three mice died post pump-implantation and were excluded from this study.

**Figure 1.**
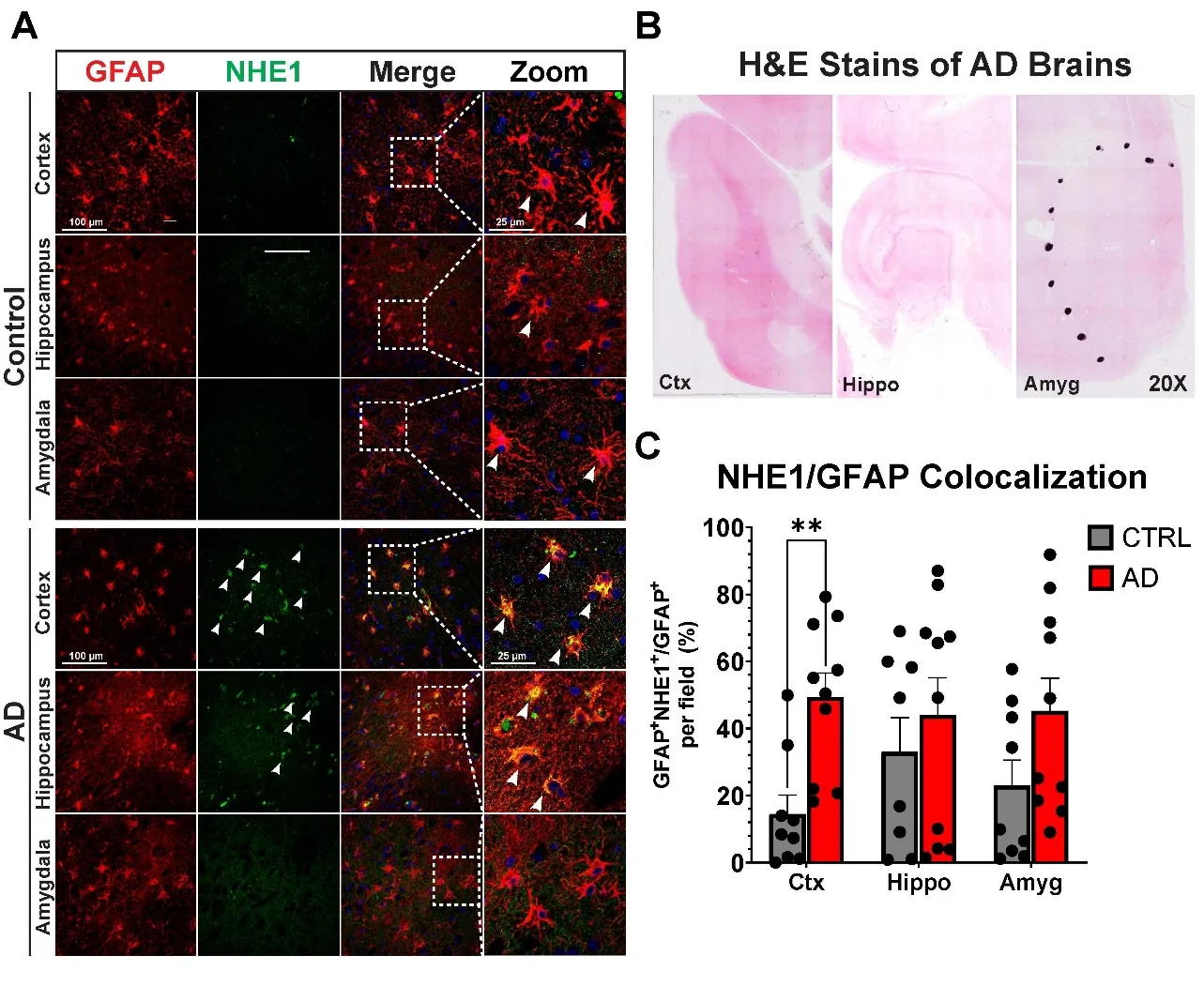
**AD post-mortem cerebral cortex displays upregulation of NHE1 protein expression in reactive astrocytes**. (A, B) Representative H&E staining and immunofluorescence staining images for NHE1 and GFAP expression in control (CTRL) and AD patient post-mortem brain tissues (cerebral cortex, hippocampus, and amygdala). Arrowheads: increased NHE1 protein localized within GFAP^+^ reactive astrocytes in different regions of AD brains than CTRL. (**C**) Summary data of NHE1^+^GFAP^+^/GFAP^+^ percentage per field in each region (**p=0.0047). Data are represented as mean ± SEM (n = 9-10 astrocyte average from 10 brains, Multiple Unpaired T-test, parametric with Sidak-Bonferroni method).

## RESULTS

### Human AD brains displayed NHE1 protein upregulation in reactive astrocytes

**Figure 2.**
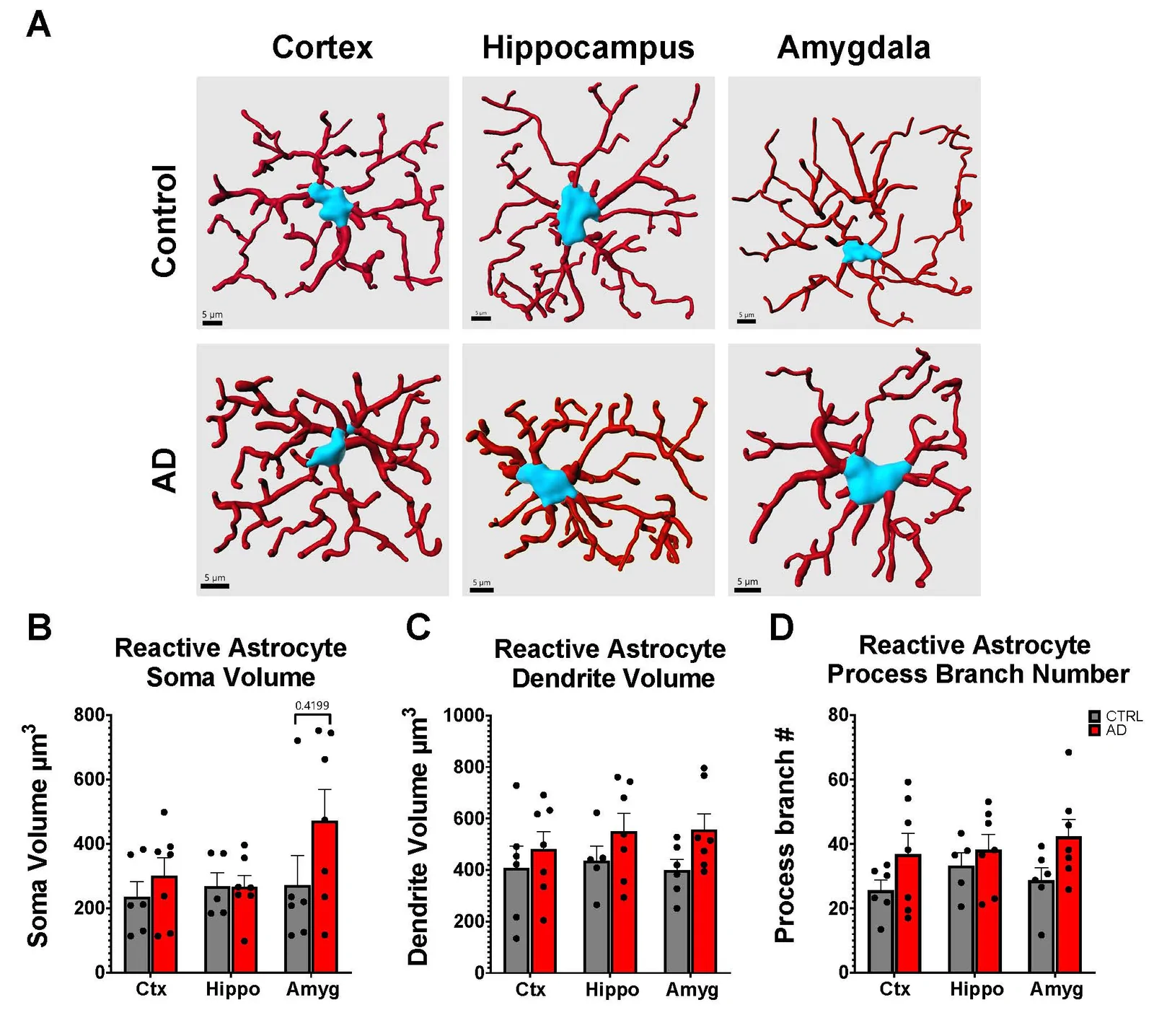
**IMARIS-rendered 3D reconstruction of reactive astrocytes in human CTRL and AD brains**. (**A**) 3D reconstructed representative images of GFAP^+^ astrocytes of CTRL and AD patient brain tissues (cerebral cortex, hippocampus, and amygdala). (**B**) Soma volume: Amygdala region difference (ns, p= 0.4199). (**C**) GFAP^+^ dendritic volume. D. GFAP^+^ process branch number. Data are represented as mean ± SEM (n=7), Multiple unpaired T-test Sidak-Bonferroni method).

We first examined whether post-mortem human brain tissues of age-matched control (CTRL) and AD subjects express different levels of NHE1 protein expression in GFAP^+^ reactive astrocytes. Detailed information of CTRL (n=9) and AD patients with Brakk Stages and comorbidities (n=10) are shown in Table 1. The AD group (including 7 males and 3 females) ranged from 64 to 97 years old with an average age of 80 and was diagnosed with 5-6 Brakk NFT stages, and 70% with mild amyloid angiopathy. The CTRL group contained 3 males and 7 females, ranged from 76 to 92 years old with an average age of 86. They were at 1-3 Brakk NFT stages and 90% of them had no amyloid angiopathy present. However, both CTRL and AD brains exhibited additional comorbidities such as aging-related tau astrogliopathy (ARTAG) or regionally specific microinfarcts. Confocal representative images of post-mortem human astrocytes expressed both NHE1 and GFAP proteins via immunofluorescence staining (Fig. 1). NHE1 expression was lower in CTRL brains but elevated in reactive astrocytes in the AD brains (specifically in cortical and hippocampal regions). The increase of the NHE1^+^/GFAP^+^ double-positive cell count was significantly higher in cerebral cortical regions of AD brains (**p < 0.005, Fig. 1C). Reactive astrocytes refer to astrocytes that undergo changes in their structure, molecular composition, and function in reaction to damage, illness, or infection within the CNS [30]. Morphological changes include hypertrophy, extended processes, altered ramification, outgrowth of long processes and increased volume of main cellular processes [31]. In assessing morphological differences of GFAP^+^ reactive astrocytes between CTRL and AD brains, we used IMARIS software to create a single astrocyte ROI for 3D reconstruction in order to discern individual astrocytes within the complex multicell-type human brain. The values for soma volume, dendrite volume and filament number of process branch points were plotted from at least 10 astrocytes for each brain section (total >90 astrocytes for CTRL subjects and AD subjects, respectively). Figure 2 shows that compared to CTRL brains, 3 regions of the AD brains (cortex, hippocampus, and amygdala) displayed an overall trend of larger soma and dendrite volumes, and increased branch numbers. Although the changes did not reach statistical significance, the trend was notable in the amygdala region of AD brains with a higher mean soma volume by 42.6% (p = 0.4199) (Fig. 2B), an increase in mean dendrite volume by 28.1% (p = 0.18) (Fig. 2C), and a higher mean process branch number by 32.3% (p = 0.19) (Fig. 2D), compared to CTRL. Altogether, the human AD post-mortem data revealed elevated NHE1 expression levels within astrocytes and that these 3D reconstructed astrocytes displayed reactive morphologies, suggesting that increased NHE1 activity could contribute to reactive astrogliosis transformation.

**Figure 3.**
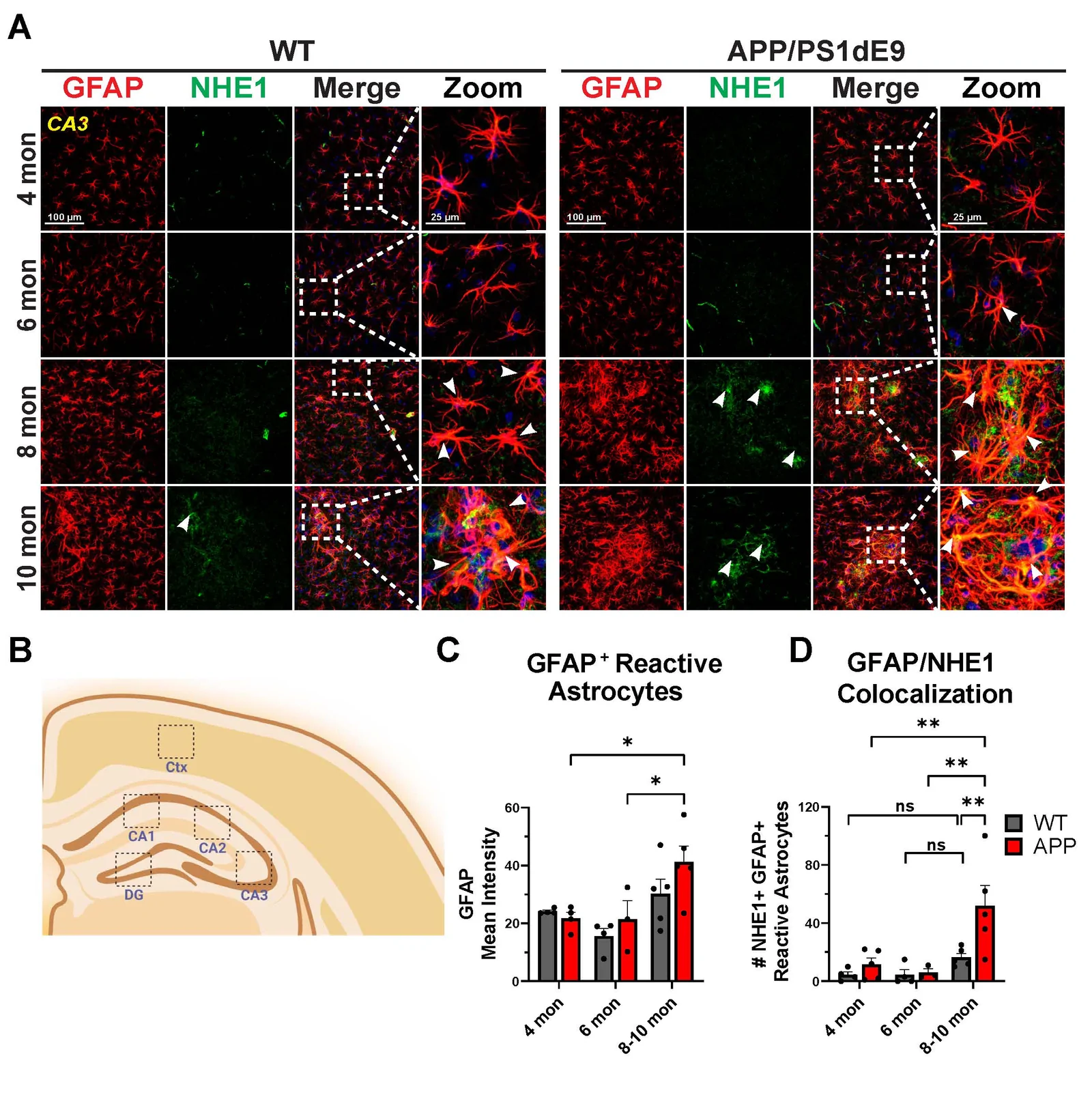
**Time-dependent elevation of NHE1 protein expression in APP/PS1dE9 reactive astrocytes**. (**A**) Representative immunofluorescence staining of NHE1 protein in GFAP^+^ astrocytes in CA3 hippocampal regions of wild-type (WT) and APP/PS1dE9 mice. Arrowhead: NHE1 expression or NHE1 expression in astrocytes. Dashed line: enlarged ROI DAPI was used for nuclear staining. (**B**) Illustration of fluorescence images acquired from hippocampal dentate gyrus, CA1, CA2, CA3 and cerebral cortex regions in WT and APP mouse brain sections. (**C**) Quantification of GFAP^+^ astrocyte mean intensity (*p<0.05). (**D**) NHE1^+^/GFAP^+^ double positive cell counts (**p<0.005) in WT and APP/PS1dE9 mice. Data are represented as mean ± SEM. (n=4-5, 2-way ANOVA, Bonferroni's Multiple comparisons test).

### Detection of NHE1 protein upregulation in GFAP^+^ reactive astrocytes in APP/PS1dE9 mice

The detection of NHE1 protein in human AD reactive astrocytes compelled us to further investigate whether the same phenotype exists in an AD mouse model. We used the well-established APP/PS1dE9 transgenic AD mouse model, in which presenilin (PS1) mutation is known to cause early onset AD and its delta E9 variant is efficient in depositing early Aβ plaques [32]. Even more, gliosis in this model was detected around 4-6 months old and cognitive impairment arising between 6-10 months [4]. We assessed changes of NHE1 protein expression in relation to GFAP^+^ astrocytes in APP mice and WT littermates at 4-, 6-, 8- and 10-months of age via immunostaining (Fig. 3A), images were taken from the cortex and four main hippocampal regions (Fig. 3B). GFAP expression in the hippocampus and cortex was significantly increased in 8-10-month-old APP mice, compared to APP mice at 4 month (*p=0.0216) and 6-month (*p=0.0348) of age, denoting a critical timepoint in reactive astrocyte development (Fig. 3C). Additionally, we observed significantly more NHE1^+^GFAP^+^ reactive astrocytes in 8-10-month-old APP mouse brains than in 8-month WT brains (*p=0.0092), or in 4-month and 6-month APP mouse brains (**p=0.0030, **p=0.0034, respectively, Fig. 3D). However, statistical comparisons between 4 mon vs 8-10mon WT brains and 6mon vs 8-10mov WT brains showed no significant differences (p>0.9999) in Figure 3D (2 Way ANOVA, Bonferroni’s Multiple Comparisons). Together, these findings echoed the human AD results and revealed an important temporal profile for reactive astrogliosis with its morphological shift and NHE1 upregulation in the mouse AD model.

**Figure 4.**
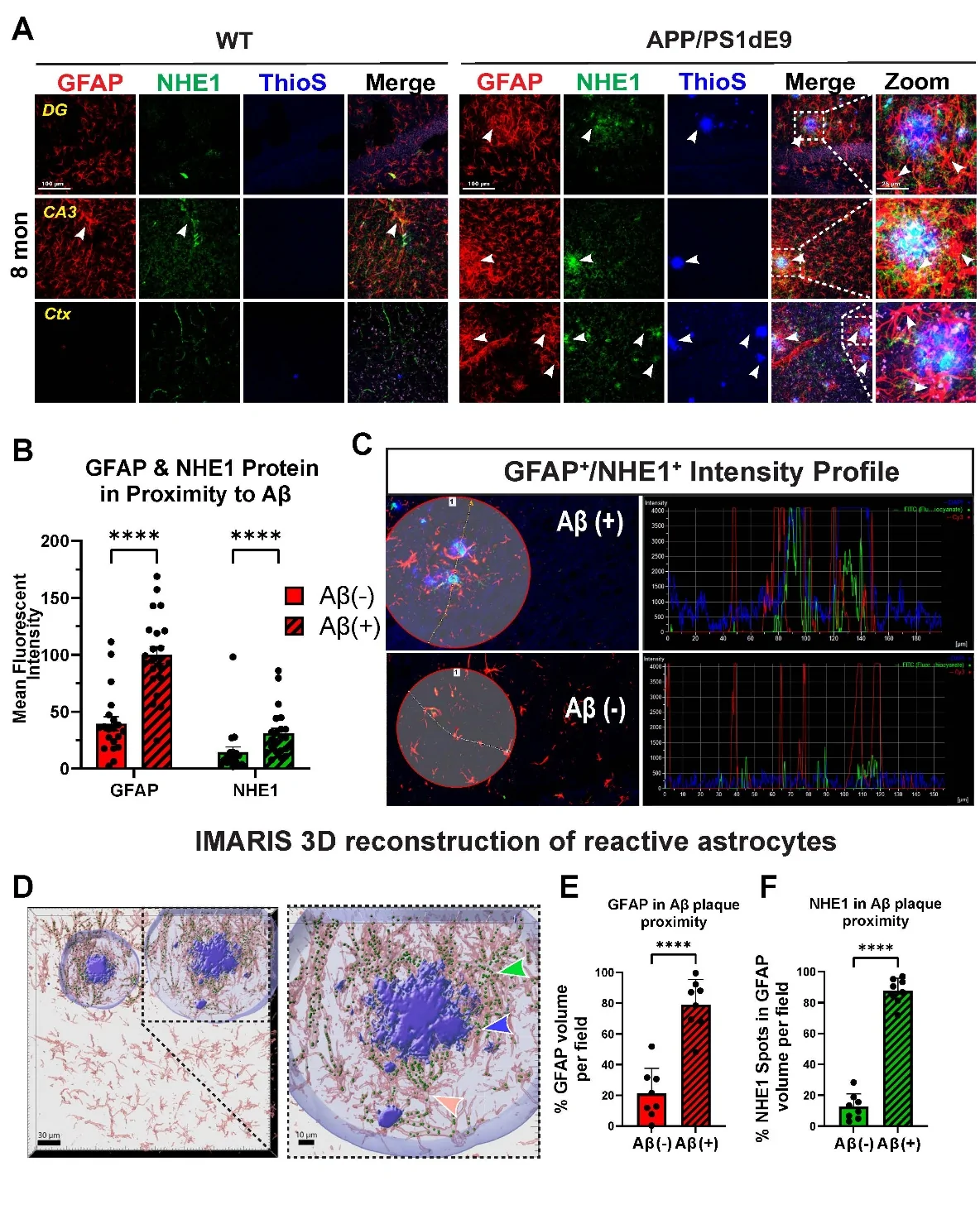
**NHE1^+^ reactive astrocytes localize in close proximity to Aβ aggregates**. (**A**) Representative confocal fluorescence images of NHE1, GFAP, and Thioflavine S-stained Aβ in WT and APP brains in dentate gyrus, CA3 and cortex regions. Arrowheads: Colocalization of NHE1, GFAP and Aβ. Dashed line: enlarged ROI displaying NHE1^+^ reactive astrocytes surrounding Aβ. (**B**) Mean fluorescence intensity of GFAP and NHE1 immunostaining in Aβ- and Aβ+ areas per field. Data are represented as mean ± SEM. (****p<0.0001, n=20, 2-way ANOVA, Bonferroni’s Multiple comparisons Test, Kolmogorov-Smirnov test for Normality used). (**C**) Intensity profile analysis of overlapping GFAP and NHE1 signals in Aβ- and Aβ+ areas with Nikon NIS Elements Analysis Software. (**D**) Representative Imaris 3D reconstruction image and zoomed image of extended surface of Aβ aggregate (blue arrow), GFAP^+^ astrocyte volume (pink arrow), and NHE1 expression spots (green arrow) in GFAP^+^ astrocytes of APP mouse cortex. (**E**) Percentage of GFAP volume per field (****p<0.0001). F. Percentage of NHE1 spots within GFAP volume per field (****p<0.0001) in Aβ+ and Aβ- areas. Data are represented as mean ± SEM (n=8, Unpaired t-test, parametric).

GFAP^+^ reactive astrocytes in the vicinity of Aβ plaques in APP/PS1dE9 mice displayed increased NHE1 protein expression

It has long been observed that reactive astrocytes interact with Aβ plaques by surrounding the plaques in an isolating “glia scar” morphology, potentially contributing to further AD pathogenesis [33, 34]. Therefore, we determined whether reactive astrocytes in close proximity to Aβ plaques also expressed elevated NHE1 expression levels in 8-10-month-old APP mouse brains [35]. WT and APP brains at hippocampal and cortical regions were assessed for GFAP and NHE1 co-expression and Thioflavine S stained Aβ plaques (Fig. 4A). Mean fluorescent intensity analysis revealed significantly higher GFAP (****p<0.0001) and NHE1 (****p <0.0001) expression in Aβ containing (Aβ(+)) regions, compared to Aβ negative (Aβ-) regions (Fig. 4B). To determine the overlap of GFAP and NHE1 immunosignals in astrocytes surrounding Aβ or away from Aβ, the intensity profiles of astrocytic GFAP and NHE1 immunostaining signals were summarized, showing the most overlap immunofluorescent intensity of GFAP and NHE1 around the Aβ(+) areas (Fig. 4C). In addition, 3D reconstruction analysis and the creation of extended plaque surfaces (Fig. 4D) illustrated a significantly higher GFAP^+^ astrocyte IMARIS rendered “surface” volume in the Aβ(+) regions per field (60%, ***p<0.0001, Fig. 4E). The percentage of NHE1 IMARIS-rendered “spots” inside of the GFAP^+^ volume was significantly higher in Aβ(+) regions (90%, ***p<0.0001, Fig. 4F). These analyses provide additional evidence that NHE1 protein elevation is not only associated with reactive astrocytes’ morphological transformation, but also could be involved in reactive astrocytes’ function in Aβ engulfment and degradation processes.

**Figure 5.**
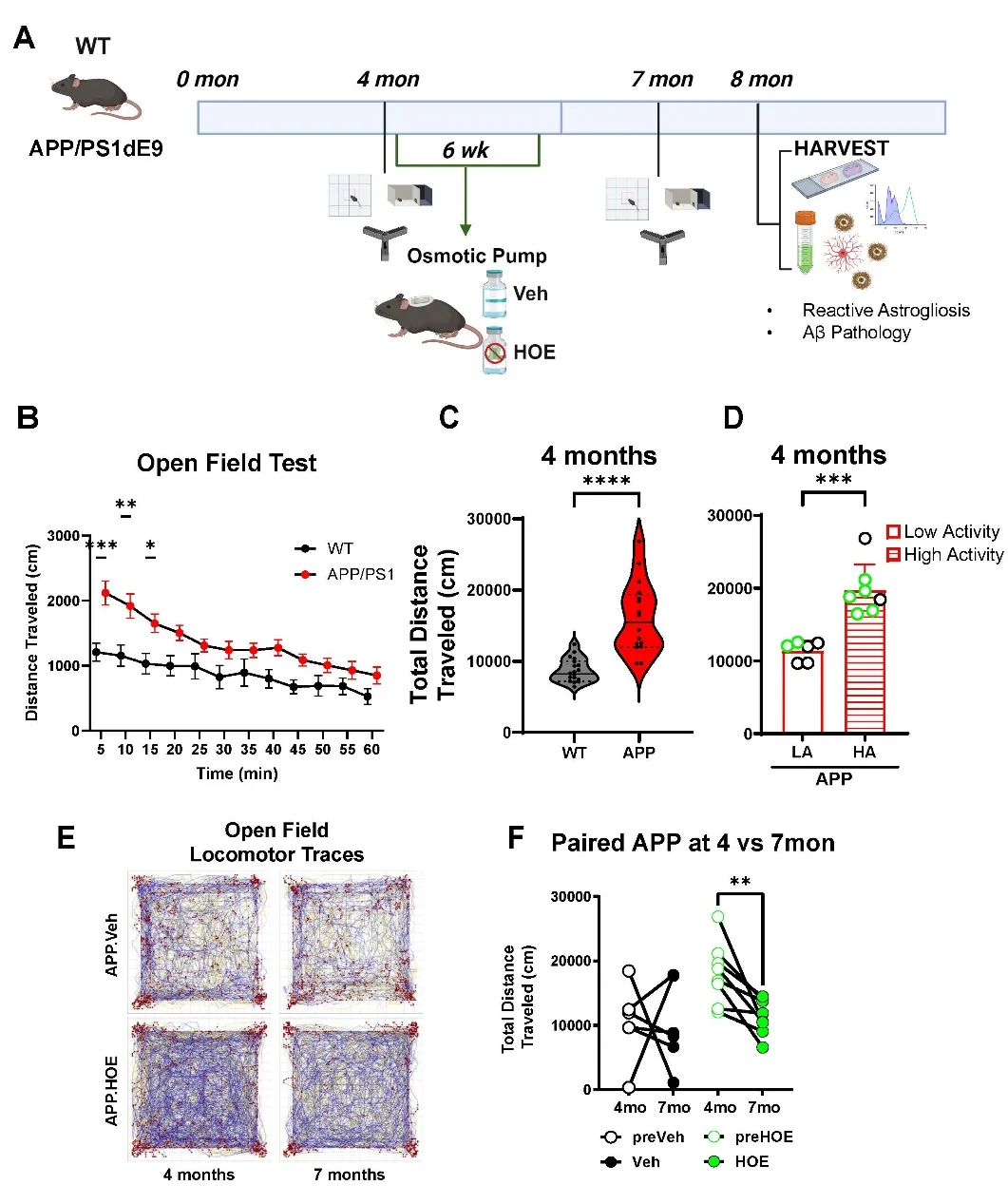
**Administration of HOE642 in APP/PS1dE9 mice decreases hyperactive locomotor activity**. (**A**) Schematic illustration of the experimental design. WT and APP/PS1dE9 mice at 4 months of age underwent baseline behavioral testing (Open Field, Light Dark, and Novel Spatial Recognition). Mice received either 6-weeks of Veh (DMSO) or HOE642 treatment via subcutaneous mini-osmotic pumps. At 8 months of age, mouse brains were harvested for reactive astrogliosis and Aβ pathology immunostaining. (**B**) Open Field Test in WT vs APP mice at 4 months of age. Lined graph displayed differences in distance traveled (*p<0.05, **p<0.005, ***p<0.001, n=16, Unpaired t-test). (**C**) Total Distance traveled in WT vs APP mice at 4 months of age (****p<0.0001, n=16, Unpaired t-test). (**D**) APP mice with low activity (LA) vs high activity (HA) based on total distance traveled (**p=0.0002, n=6, Unpaired t-test, parametric). (**E**) Total distance locomotor representative traces of APP.Veh vs APP.HOE mice at 4 and 7 months of age. (**F**) Pairwise comparison of total distance traveled in APP.Veh and APP.HOE mice at 4 vs 7 months of age (**p=0.0065) (n=8, Paired t-test). All Data are represented as mean ± SEM.

### HOE642 administration in APP/PS1dE9 mice attenuated locomotor hyperactivity development

We hypothesized that NHE1 protein elevation during reactive astrogliosis may contribute to AD pathogenesis and neurological behavior deficits. The APP/PS1dE9 mouse line has been reported to display early pathological changes with hyperactive locomotor behavior at 4-6 months of age and followed by later cognitive decline around 6-7 months of age [32, 35]. Therefore, efficacy of pharmacological blockade of the NHE1 protein was assessed in the early AD pathogenesis stage by administering Veh or HOE642 at 4 months of age (Fig. 5A). Four cohorts of mice (WT.Veh, WT.HOE, APP.Veh, APP.HOE) received either Veh or HOE *in vivo* for 6 weeks via subcutaneous mini-osmotic pump implantation. The Open Field Test (OFT), Light/Dark (LD) and the Novel Spatial Recognition (NSR) tests were conducted in mice at 4 months of age prior to the treatment regimen for baseline neurobehavioral assessment and again at 7 months of age for post-treatment assessment as illustrated in Figure 5A. Together, these tests evaluated limbic system function, motor behavior, anxiety-like behaviors, and hippocampal and medial temporal lobe contributions to spatial learning and memory [29]. At 4 months of age baseline assessment, APP mice displayed a significant increase in distance traveled during the first 15 minutes of the OFT (***p<0.001) (Fig. 5B) as well as overall total distance travel over the entire 60 minutes of the test (****p<0.0001) (Fig. 5C), compared to WT littermates. There were no differences in vertical activity counts and perimeter time in the OFT between APP mice and the WT littermates (Supplementary Fig. 1 Behavior). Based on recent reports on hyperactive locomotor behavior displayed in this APP mouse line [35], we also identified two subgroups of APP mice at 4-months old that displayed either normal distance travel activity (LA) or a more hyperactive locomotor activity phenotype (HA) (**p=0.0002, Fig. 5D). This phenotype could partially be related to the Hyperactivity-Impulsivity-Irritiability-Disinhibition-Aggression-Agitation (HIDA) behaviors commonly described in AD mouse models and AD clinical research [4, 35, 36]. The total distance traveled field traces from APP.Veh mice illustrate that locomotor behavior trends remained unchanged from 4 to 7 months of age, but after 6-weeks of HOE treatment, the hyperactive locomotor behavior observed in the APP.HOE mice at 4-months of age had notably lessened by 7-months of age (Fig. 5E). The pairwise comparison analysis of the APP.Veh and APP.HOE groups from 4 to 7 months of age further illustrated that only the APP.HOE mice showed significantly decreased total distance travel at 7 months of age from the 4 months of age (**p=0.0065) (Fig. 5F). These results suggest that pharmacological inhibition of NHE1 activity attenuates hyperactive striatal locomotor activity in the APP/PS1dE9 mouse model.

### Administration of HOE642 in APP/PS1 mice does not affect anxiety or short-term spatial memory behaviors

**Figure 6.**
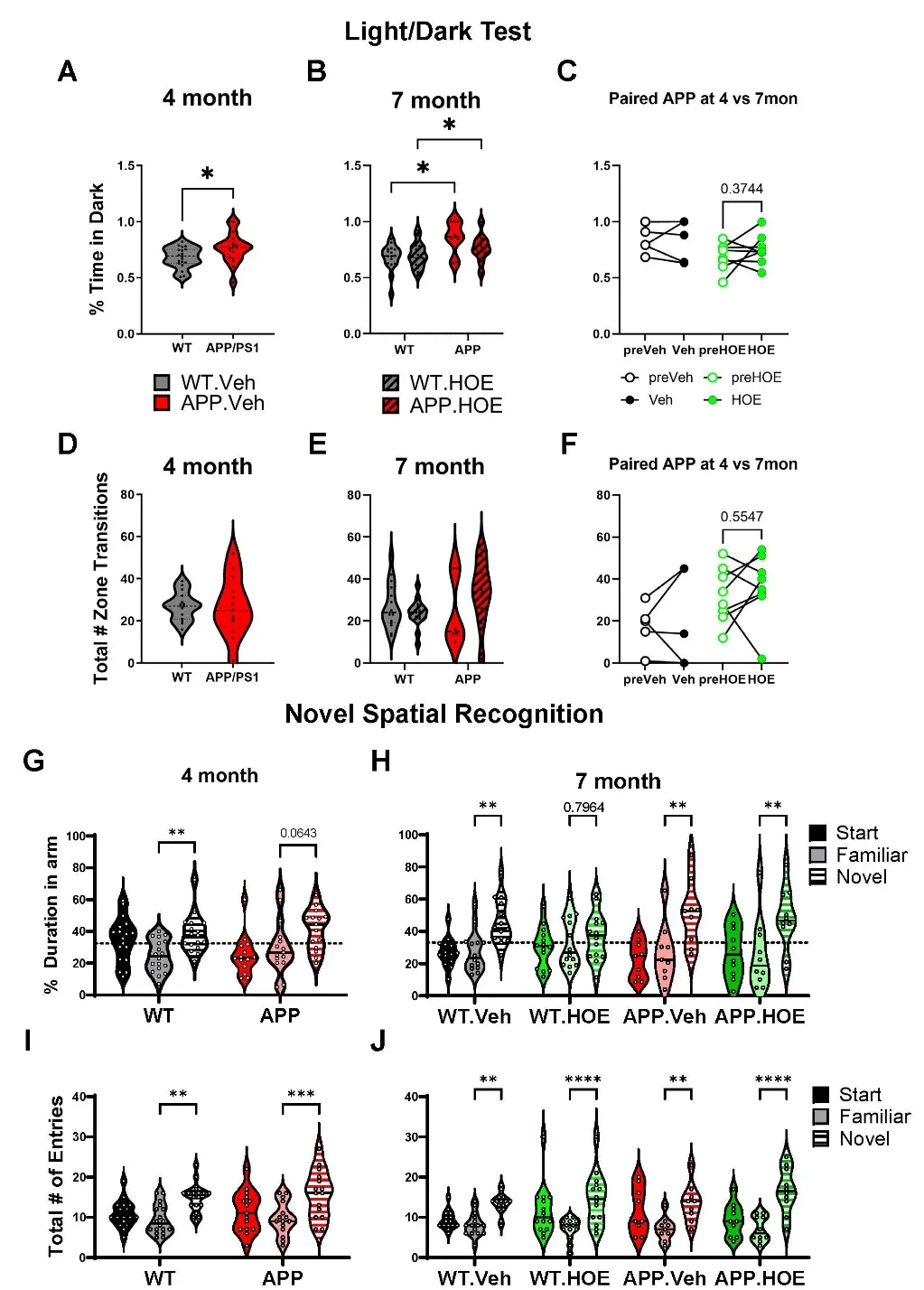
**Administration of HOE642 in APP/PS1 mice does not affect anxiety or short-term spatial memory behaviors**. (**A**) Percent time spent in the dark zone in WT and APP mice in Light Dark Test at 4 months of age (*p=0.0345, n=17-19, Unpaired t-test). (**B**) Comparison of percent time spent in dark for WT.Veh, WT.HOE, APP.Veh, and APP.HOE mice at 7 months of age (*p=0.0102, n=12, 2-Way ANOVA, Bonferroni's Multiple Comparisons). (**C**) Pairwise comparison of time spent in the dark zone in APP.Veh and APP.HOE mice at 4 vs 7 months of age (ns, p=0.3744, n=8, Paired t-test). (**D**) Total # Zone Transitions in Light Dark Test in WT vs APP mice at 4 months of age (ns, p=0.9145, n=13, Unpaired t-test). (**E**) Comparison of total # zone transitions for WT.Veh, WT.HOE, APP.Veh, and APP.HOE mice at 7 months of age (WT.V vs APP.V, p>0.9999 and WT.HOE vs APP.HOE, p=0.2675 )(ns, n=12, 2-Way ANOVA, Bonferroni’s Multiple Comparisons.) (F) Pairwise comparison of transition # in the dark zone in APP.Veh and APP.HOE mice at 4 vs 7 months of age (ns, p=0.5547, n=7, Paired t-test). (**G**) Percent time spent in novel arm compared to familiar arms for WT and APP mice in Novel Spatial Recognition task at 4 months of age (WT, **p=0.0074 and APP, ns, p=0.0643, n=18). (**H**) Percent time spent in novel arm compared to familiar arms for WT.Veh (**p=0.0057), APP.Veh (**p=0.0030) and APP.HOE (****p<0.0001) at 7 months (n=10-14). (**I**) Number of entries in novel arm for 4-month WT (**p=0.0010) and APP mice (***p=0.0007, n=16). (**J**) Number of entries in novel arm at 7 months for WT.Veh,(**p=0.0034), WT.HOE (****p<0.0001), APP.Veh (**p=0.0060), and APP.HOE (****p<0.0001) mice (n=16). All data are presented as mean ± SEM (G-J, 2-Way ANOVA Bonferroni’s Multiple comparisons test.)

APP mice at 4 months of age displayed anxiety like behaviors in the Light Dark (LD) Test and spent significantly more time in the dark area than WT mice (*p=0.0345) (Fig. 6A). This phenotype in APP.Veh group persisted at 7 months of age, compared to WT-Veh mice (**p=0.0102). Similarly, APP.HOE mice at 7 months of age spent significantly more time in the dark zone than the WT.HOE treated mice (**p=0.0102, Fig. 6B). However, HOE treatment did not significantly change the time APP mice spent in the dark zone from 4 to 7 months of age in the pairwise comparisons (ns, p=0.3744, Fig. 6C). There were no significant differences in the number of zone transitions in the LD test between WT and APP mice at 4 months of age (Fig. 6D) or at 7 months of age (Fig. 6E). Pairwise comparisons of zone transition numbers between APP.Veh and APP.HOE mice from 4 to 7 months displayed no significant differences in entries into the dark zone (ns, p=0.5547) (Fig. 6F). Given the above results, we concluded that HOE642 treatment did not make significant changes in anxiety-like behaviors in APP.HOE mice from 4 to 7 months.

We further determined if NHE1 protein inhibition altered short-term spatial learning memory in APP mice from 4 to 7 months using the Novel Spatial Recognition (NSR) test. At 4 months of age, both WT and APP mice displayed normal spatial memory, with significantly more total time (> 33%) spent in the novel arm, indicated by the dashed line, and when specifically compared to time spent in the familiar arm (WT, **p= 0.0074 and APP, p=0.0643) (Fig. 6G) as described by Rizzo et al, [29]. These results suggest intact short-term spatial memory in APP mice at 4 months of age. By 7 months of age, all cohorts of mice spent more than 33% of the total time in the novel arm, while only 3 groups spent significantly more time in the novel arm than the familiar arm, WT.Veh (**p=0.0057), APP.Veh (**p=0.0030) and APP.HOE (**p=0.0047) (Fig. 6H). WT and APP mice at 4 months displayed higher number of total entries into the novel arm compared to the familiar arm (WT, ***p=0.0010 and APP, ***p=0.0007) (Fig. 6I). By 7 months, all groups entered the novel arm significantly more times than the familiar arm (WT.Veh **p=0.0034, WT.HOE ****p<0.0001, APP.Veh **p=0.0060, APP.HOE ****p<0.0001) (Fig. 6J). Taken together, the NSR test results indicate that our cohort of APP mice at 4 and 7 months of age exhibit intact short-term spatial memory function, similar to WT mice. Furthermore, we concluded that pharmacological inhibition of NHE1 protein with HOE has no enhancing or detrimental effects on the cognitive memory functions by 7 months of age. Moreover, the significant number of entries into the arms in APP mice may result from hyperactive locomotor behavior, as observed in the Open Field Test, rather than from the enhanced spatial memory.

**Figure 7.**
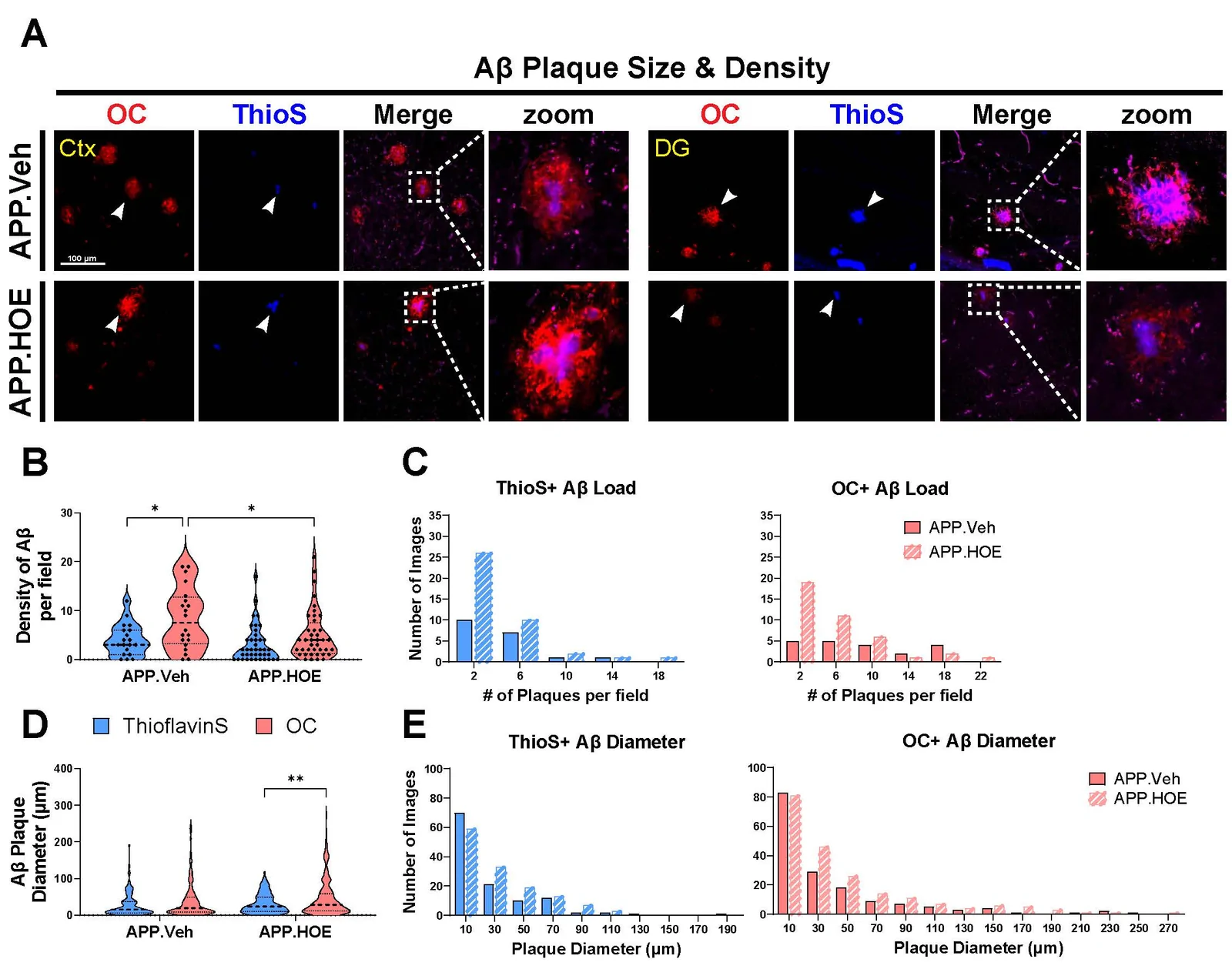
**Administration of HOE642 in APP/PS1 mice reduces OC+ Aβ plaques density**. (**A**) Representative 40x images of Aβ plaques stained with OC and Thioflavin S in cortex (Ctx) and hippocampal dentate gyrus (DG) regions of 8-month-old APP.Veh and APP.HOE mice. Dashed line: enlarged ROI displaying OC+ amyloid fibrils surrounding ThioS+ Aβ plaques. Arrow Heads: Individual OC+ and ThioS+ plaque occurrences. (**B**) Quantification of Thio S+ and OC+ Aβ plaque densities per field between APP.Veh and APP.HOE brain regions, APP.Veh ThioS+ vs APP.Veh OC+ (*p=0.0305), APP.HOE ThioS^+^ vs APP.HOE OC^+^ (*p=0.0299), APP.Veh OC^+^ vs APP.HOE OC^+^ (*p=0.0380) with non-parametric, Mann-Whitney Multiple t-test (n= 20 plaques from a total of 4 brains). Dashed Line: median of data. Dotted Line: Quartiles of Data. (**C**) Frequency distribution histogram of # of Aβ plaques per 40x field in Ctx and DG. (**D**) Quantification of ThioS^+^ and OC^+^ Aβ plaques diameters (µm) in APP.Veh and APP.HOE brains, APP.HOE ThioS^+^ vs APP.HOE OC^+^ (n=120 plaques from a total of 4 brains, *p=0.0432, non-parametric, Mann-Whitney Multiple t-test). Dashed Line: median of data. Dotted Line: Quartiles of Data. (**E**) Frequency distribution histogram of Aβ plaque diameters. All data are represented as mean ± SEM.

### HOE642 administration in APP/PS1dE9 mice attenuates OC-recognized Aβ fibril accumulation

Targeting reactive astrocytes via astrocyte function modulators in mouse AD models has been shown to lessen AD brain pathology and AD-induced cognitive decline [4]. In this study, we tested the efficacy of NHE1 inhibitor HOE642 on attenuation of astrogliosis and Aß aggregate size, and density in 8 months old APP mice. Aß plaque morphology and characteristics were assessed by staining with both OC antibody and Thioflavin S (Thio S) dye, which binds to Amyloid fibrils and mature Aβ plaque dense core structures, respectively, [34, 37, 38]. As shown in Figure 7A, confocal images illustrate Aß in APP.Veh and APP.HOE cortex (Ctx) and dentate gyrus (DG) brain regions, in which OC probes for dispersed amyloid fibrils made of highly aggregated Aβ (arrowhead) and surrounds the ThioS stained densely compact core of mature Aβ plaques (arrowhead). Quantification of the overall density of ThioS+ Aβ plaques and OC+ Aβ plaques in hippocampal and cortical regions show that APP.Veh brains displayed significantly higher OC+ Aβ plaque density per field, compared to APP.Veh ThioS+ Aβ plaques (*p=0.0305). A significant difference between OC+ and ThioS+ Aβ stain was also observed in the APP.HOE brains (*p=0.0299). In addition, the density of OC+ Aβ plaques was significantly decreased in the APP.HOE brains, compared to the APP.Veh brains (*p=0.0380) (Fig. 7B). No differences in the density of ThioS+ Aβ plaques were detected between the APP.Veh and APP.HOE brains. Moreover, frequency distribution histograms of Aβ density illustrated that a lower number of ThiosS+ or OC+ Aβ plaques per field are more frequently detected in the APP.HOE brain images (Fig. 7C). Figure 7D shows that there were no significant differences in Aβ diameters in the APP.Veh brains when stained with ThioS dye or OC amyloid fibril antibody. However, the APP.HOE brain exhibited significantly larger OC+ Aβ diameter than ThioS+ Aβ plaques (*p=0.0432). Similar distribution of ThioS+ and OC+ Aβ plaque diameters were detected between the APP.Veh and APP.HOE brains (Fig. 7E). Taken together, our data suggest that HOE642-mediated NHE1 inhibition attenuates Aβ fibril aggregates, specifically detected by the OC Aβ fibril peptide antibody.

## DISCUSSION

### Astrogliopathology in AD brains and changes of NHE1 protein in GFAP^+^ astrocytes

AD patients exhibit reactive astrogliosis at earlier stages of pathogenesis and gradually transition into astrocyte atrophy at later stages of AD [39]. However, before astrocytes reach this atrophy stage, much of the underlying AD pathological dysfunction has already been initiated. Studies of biofluid samples from early-stage AD patients reveal correlations between increased GFAP and abnormal levels of CSF Aβ [40]. In AD brains, reactive astrocytes with altered transcriptome profiles morphologically transform to surround Aβ deposits and to release inflammatory factors in attempt to digest accumulated Aβ deposits [41]. Astrocytes surrounding Aβ plaques are assumed to be in a semi or intermediate reactive state, initially promoting neuroprotection, as seen in subclusters of more homeostatic astrocytes in recent astrocyte transcriptomic studies of AD brains [42, 43]. On the contrary, the more detrimental reactive astrogliosis response is hypothesized to contribute to AD pathogenesis, not solely because of morphological changes, but more so due to transcriptional and functional changes that accompany the astrocyte structural changes [15, 39, 41, 42].

Numerous studies have revealed spatially different astrocytic subclusters within healthy and diseased brains with specific genetic profiles, suggesting astrocyte subtypes with varying homeostatic roles and functions [1, 42, 44]. Single-cell RNA sequencing data of LPS-induced cortical astrocytes revealed one cluster with a gene signature displaying increased metalloproteinase inhibitor *Timp1* whose expression in astrocytes is associated with Aβ response in AD [44]. Another cluster of astrocytes was confirmed in the 5xFAD mouse model of AD with gene signature for astrocytes’ involvement in interferon response with high Trem2 and Igtp expression, located near regions with high immune cell density in spatial transcriptomics analysis [44]. Single-nuclei data on astrocytes from APOE ε2/3 human AD brains showed both common and cluster-specific changes in gene signatures of AD astrocytes, including upregulation in genes such as HPE2, a heparinase homolog antagonizing heparinase enzymatic degrading activity, and NEAT1, nuclear enriched abundant transcript 1, whose inhibition could actually help promote Aβ deposit removal [43]. However, our understanding of astrocyte specific transcriptional and functional dysregulation in AD pathology remains incomplete.

In this study, we investigated both morphological and functional changes of reactive astrocytes in AD pathogenesis. Probing for GFAP, the astrocyte’s intermediate filament of the cytoskeleton, is commonly utilized for detecting morphological changes in reactive astrocytes. Using IMARIS 3D rendering of brain slice images, we detected increased GFAP^+^ astrocyte volume in the proximity of Aβ plaques in APP mouse astrocytes, consistent with the reactive astrocyte response at the neuroinflammatory state which can further exacerbate the inflammatory response [45]. We also found increased NHE1 protein expression (a ratio of NHE1 spots to GFAP^+^ volume) in reactive astrocytes in proximity of the plaques, compared to astrocytes away from the plaques. This increase in NHE1 protein within reactive astrocytes could denote a response to reactive oxygen species, inflammation and/or pH dysregulation [46]. Anti-mouse NHE1 antibody (Santa Cruz #sc-136239) has been validated in astrocyte cultures with siRNA of NHE1 (Supplementary Fig 2). In future studies, *in situ* RNA scope probing for NHE1 protein gene *Slc9Ac* and localization in GFAP^+^ and other astrocyte subpopulations would provide insight as to whether NHE1 gene is upregulated in specific clusters of astrocytes during AD disease progression.

### Pharmacological NHE1 inhibition on astrogliosis, neuropsychological function and Aß aggregate accumulation

Our recent studies in vascular dementia and ischemic stroke models demonstrated that blocking NHE1 protein activity either by pharmacological inhibition or astrocyte specific NHE1 gene knockout attenuates astrogliosis, neurodegeneration, and cognitive decline [17, 20]. In this study, we tested whether pharmacological NHE1 protein inhibition can attenuate AD-induced social, locomotor and memory deficits, astrogliosis and Aβ pathology in the transgenic APP/PS1dE9 mouse model. Astrogliosis and Aβ pathology at 4-6 months of age in the APP mice were accompanied with hyperactive and anxiety like behaviors in the OFT or Light/Dark assays, respectively. The hyperactive phenotype in the APP/PS1 model at 4 months of age has previously been observed on different background strains, for example the B6 APP/PS1 transgenic line consistently displayed hyperactivity in total distance travelled in the OFT [47]. Wang and colleagues attributed observed locomotor hyperactivity in 4-month-old APP/PS1 mice to astrocytic glymphatic impairment as a result of aquaporin 4 mislocalization as well as Aß load burden in the motor cortex [35]. Whether this hyperactive behavior is a sustained phenotype in APP mice at 7 months was unknown. Using a pairwise comparison in the OFT, we revealed that there were no significant differences in distance traveled in the APP.Veh mice from 4 to 7 months of age. In contrast, a significant decline in overall distance traveled in the APP.HOE mice was detected at 7 months of age, compared to 4 months of age prior to the HOE642 treatment. HOE642 also significantly attenuated the overall density of OC+ Aβ peptide fibrils in the APP.HOE mouse cortical and hippocampal brain regions at 8 months of age. Taken together, our results demonstrate that pharmacological inhibition of NHE1 protein at the early stage of AD attenuates hyperactivity behavior and reduces Aβ pathologies.

Clinically, in addition to dementia, AD patients are often diagnosed with other behavioral and psychological symptoms and these behaviors normally consist of aggressiveness, irritability, and hyperactivity, referred to as the HIDA domain by Keszycki and colleagues [36]. Many of these symptoms are associated with the general prefrontal cortex gray matter loss and specific molecular loss of corticostriatal control and monoaminergic pathways [36]. The neurodegeneration of frontal cortical areas to ventral and dorsal areas could lead to the presentation of HIDA domain behaviors. Early astrocyte Ca^2+^ dysfunction has been suggested to play a role in AD pathogenesis prior to the appearance of Aβ pathology and major cognitive symptoms [48, 49]. Shah and colleagues reported that disrupted astrocyte Ca^2+^ signaling contributes to elevated functional connectivity in the anterior cingulate cortex in AD patients and in APP^NL-F^ mouse model [49]. NHE1 protein-mediated Na^+^ influx can lead to elevation of intracellular Ca^2+^ via stimulating reversed operation of Na/Ca exchanger. Attenuating hippocampal astrogliosis in traumatic brain injury with NHE1 inhibitors EIPA and amiloride may be due to blocking Na/Ca exchanger-mediated Ca^2+^ influx [50-52]. Whether the attenuated motor hyperactivity in the APP.HOE mice involve indirect inhibition of Na/Ca exchanger activity in astrocytes or neurons remains to be elucidated.

### Role of astrocytic NHE1 protein in Aβ accumulation

In most neurodegenerative diseases, proper degradation of misfolded and aggregated protein fragments such as Aβ, relies heavily on phagocytosing microglia and astrocytes, along with their intracellular degradation machinery such as highly acidic lysosomes [19, 53]. Lysosomal acidification is maintained by V-ATPase, an enzymatic proton pump complex responsible for maintaining lysosomal ~ 4-4.5 pH by pumping protons into the lysosome against their intracellular concentration gradient [54]. This physiological lysosomal pH is essential for the activation of lysosomal enzymes such as cathepsins and hydrolases to efficiently degrade unwanted intracellular debris and misfolded proteins [25]. However, the deacidification of lysosomes impairs their ability to fuse with the autophagosome and prevents proper degradation and clearance of Aβ [53]. Recent findings report more alkaline and dysfunctional lysosomes in PS1-FAD mutation human fibroblasts, APOE3 & 4 IPSC iAstrocytes and neurons of 5xFAD, Tg2576, TgCRND8, PSAPP, and APP51 AD mice [25, 55, 56]. Moreover, lack of phosphorylation of PS1 at the Ser376 site in 5xFAD and in PS1^KI/KI^ AD mouse microglia prevents proper recruitment of V-ATPase ATP6V0a1, leading to proton pump dysfunction and deacidification of lysosomes [19, 57].

**Figure 8.**
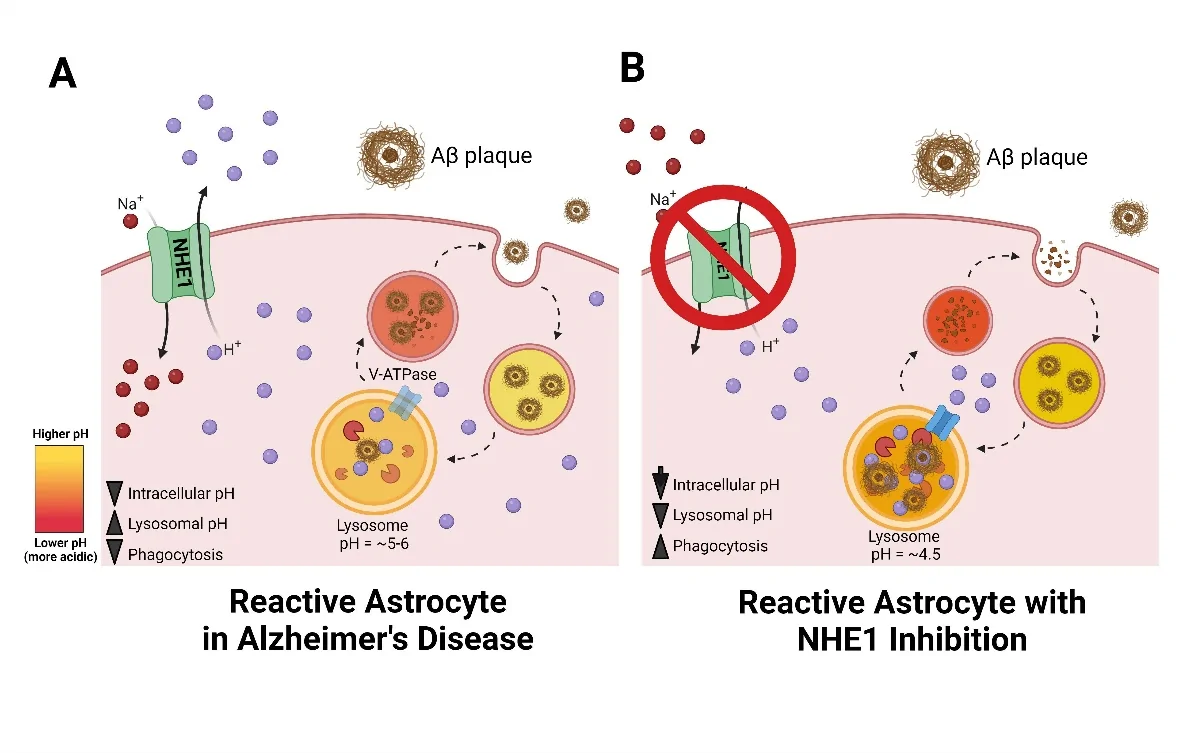
**Schematic illustration of NHE1 inhibition on reactive astrocyte intracellular pH regulation and lysosomal-mediated Aβ plaque degradation**. (**A**) NHE1 upregulation in APP/PS1dE9 mouse reactive astrocyte may increase H^+^ efflux and Na^+^ influx, creating a more alkaline intracellular pH. This subsequent lower H^+^ concentration could, indirectly, further exacerbates impaired V-ATPase pump activity reported in APP/PS1dE9 brains [19, 25]. This leads to reduced acidification of lysosomal pH and a decrease in proper Aβ protein degradation. (**B**) When NHE1 protein is inhibited with HOE642, NHE1-mediated H^+^ efflux is blocked and intracellular pH is reduced, maintaining a higher intracellular H^+^ concentration, which favors V-ATPase to pump H^+^ into lysosomes, indirectly enhancing proper lysosomal acidification and degradation of Aβ fibrils and plaques.

In the context of AD, the mechanisms underlying NHE1 protein upregulation and beneficial effects of using inhibitors such as HOE642 are not clear, yet we speculated two possible mechanisms. Since we detected significant decrease in the overall density of Aβ peptide fibrils in the APP.HOE brain, we hypothesized that this outcome may result from blocking NHE1-mediated H^+^ efflux and acidification of the pHi, which facilitates V-ATPase-mediated proton uptake back into the lysosome, restoring proper lysosomal pH acidification in the APP.HOE brains and degradation of extracellular debris and Aβ misfolded proteins (Fig. 8). We speculate that astrocytic as well as microglial lysosomes can degrade the looser form of Aβ peptide deposits before they form the densely compact form of Aβ aggregate deposits which are detected by Thioflavin S dye. Further investigation of Aβ plaque degradation by microglia and astrocytes in the APP.HOE brains is warranted.

In regards to this Aβ and lysosomal interplay, several studies support the role of intracellular Aβ exposure in impairing lysosomal acidification and proper degradation [53, 58]. The accumulation of Aβ aggregates results in V-ATPase subunit dysfunction, which reduces lysosomal H^+^ and therefore induces a more alkaline lysosomal environment. Moreover, the A246E amino acid mutation in the PS1 subunit leads to reduced assembly of V-ATPase, thus, both accumulated Aβ and PS1 mutation in APP/PS1dE9 mouse astrocytes could lead to leaky lysosomal H^+^ causing an acidic pH_i_. Thus, elevated NHE1 protein expression in reactive astrocytes may indicate a cellular response in attempting to counteract cytosolic acidosis and to restore the physiological pH_i_ homeostasis as we hypothesize from our results in decreased OC+ Aβ fibril density. Interestingly, down-regulation of endolysosomal NHE6, NHE9 and V-ATPase V0a1 subunit transcripts were all detected in ApoE4 AD astrocytes, which significantly exacerbates acidification of endosomal pH and deacidification of lysosomal pH detected by pH sensitive probes and FACS, suggesting that intracellular, lysosomal, and endosomal pH are affected in the context of AD gene related mutations [59].

In our second hypothesis, we speculate that blocking NHE1-mediated H^+^ efflux with HOE642 treatment in APP/PS1dE9 mice may also restore acidification of lysosomal pH through a mechanism of available energy sources. The availability of ATP, which as an energy source, plays a major role in maintaining proper lysosomal acidification and a more general role in hypometabolic AD pathogenesis [60]. The conversion of ATP to cAMP by soluble adenyl cyclase works in the acidification of lysosomes as V-ATPase relies on this energy source to pump protons into this organelle against its concentration gradients [58]. Blocking NHE1 protein activity significantly stimulates more mitochondrial oxidative phosphorylation than glycolysis [17, 61, 62]. Thus, HOE642 treatment could improve cytosolic and endosomal pH homeostasis by increasing H^+^ as well as ATP supply for V-ATP-mediated lysosomal acidification. However, we cannot rule out other pH regulatory mechanisms in the regulation intracellular and lysosomal pH in AD astrocytes. Another important pH regulator in astrocytes is NBCe1, which pumps Na^+^ and HCO_3_ at a 1:2 ratio either at an inward or outward direction. Giannaki and colleagues (2021) reported that increased NBCe1 expression and activity was able to restore proper astrocytic pHi in highly acidic/basic *in vitro* environments by modulating STAT3 transcription and GFAP expression in cortical and hippocampal astrocytes [63]. Exposure to Aβ and tau aggregates led to NHE6 mediated autophagic dysfunction at the endo-lysosome causing neurodegeneration [64]. Future studies using scRNAseq transcriptomics could gain a wider view of gene networks involved in lysosomal dysregulation and impact of pharmacological blockade of NHE1 protein.

### Off-target effects of NHE1 inhibitor and clinical translation potential

HOE642 (Cariporide) was developed as an NHE1 selective inhibitor and has been extensively studied in experimental animal research and clinical trials [65]. NHE1, encoded by the Slc9a1 gene, is from a family of 10 NHE isoforms that functions to regulate intracellular pH, osmolarity and cell volume, proliferation, and intracellular trafficking [66]. It has been reported that the IC50 values of Cariporide for expressed human NHE1 (hNHE1), hNHE2, and hNHE3 isoforms are 30nM, 4.3nM, and over 100 µM, respectively [67]. These NHE isoforms could be potential off targets of HOE642. Of the NHE isoforms, NHE1 is most abundantly expressed in the brain and localized to areas of the hippocampus and cortex while other isoforms are highly expressed in other organs such as the stomach, kidneys, and intestines [68]. NHE2 and 3 are less abundant in the brain cells, with NHE3 being detected in cerebellar Purkinje cells [69, 70]. We speculate that administering HOE642 via mini-osmotic pump (at a concentration of 0.3mg/kg) is mainly blocking NHE1 protein activity. However, NHE1 gene is considered as a “house-keeping” gene for its various functions [71] and is ubiquitously expressed in all cell types in the CNS and throughout the rest of body [72]. Global knockout of NHE1 in mice resulted in a wide range of physical impairments and cognitive deficits, especially epileptic seizures and overall increased neuronal excitability [72]. Administering HOE642 subcutaneously would globally inhibit NHE1 protein activity. However, we did not detect any significant differences in animal survival, motor function, or neurological spatial memory between WT.HOE and WT.Veh mice, implying a minimum systemic off-target effects of HOE642. But we cannot rule out that the HOE642 effects in APP.HOE mice could also include blocking NHE1 protein activity in other brain cells such as neurons, and microglia, etc. Future studies with astrocyte specific NHE1 knockout approach in APP/PS1dE9 mouse line will benefit in evaluating impact of astrocytic NHE1 inhibition on early AD pathology.

HOE642 had been previously tested in clinical trials for reducing myocardial infarction in the ESCAMI, GUARDIAN, and EXPEDITION studies [66, 73]. The EXPEDITION study targeted patients undergoing coronary artery bypass surgery (CABG) and employed continuous infusion of Cariporide starting at higher doses [74]. This study revealed protective effects against nonfatal myocardial infarction but reported incidents of death mostly attributed to thrombotic stroke events and potential platelet dysregulation [66, 75]. Efficacy of chronic inhibition of NHE1 protein via pharmacological approaches has not been extensively studied. In a golden retriever model of muscular dystrophy, NHE1 inhibitor Rimeporide has shown protective effects on left ventricle ejection fractions and reduced overall left ventricle deterioration [76]. In our study, 6-week administration of HOE642 was tolerated in both WT and APP mice. Testing efficacy of HOE642 or new NHE1 inhibitors in aged APP/PS1 mice or other AD animal models is warranted to assess their clinical translation potentials.

### Limitation of the study

Several experimental limitations arose throughout the execution of this study. To render improved 3D reconstruction of human brain slices, thicker slices of pre-mounted post-mortem tissues will be required for more accurate three-dimensional depth analysis in future studies. Another optimization would be accurately reconstructing the soma of astrocytes. For our astrocyte nuclear volume analysis, we only acquired data using the TRITC GFAP channel because DAPI nuclear staining were less morphologically uniform and less consistent than what we could pull from our red GFAP staining channel. We partially addressed this issue by merging both TRITC & DAPI channels, which allowed better differentiation between DAPI stained nuclei and TRITC stained astrocyte somas. Additional Scholl analysis for branching quantification of reactive astrocytes could alternatively be utilized with Image J, however, we found IMARIS to offer more parameters. In this study we utilized GFAP antibody, our primary astrocytic marker, but we understand that GFAP does not efficiently label all elements of astrocytes. Many other protein markers such as aldehyde dehydrogenase family 1, member 1; aquaporin 4 water channel; calcium-binding protein S100β; or glutamate transporters solute carrier family 1 member 2 are often used to label various aspects of astrocytes [77]. They provide information on astrocytes glymphatic dysfunction, astrocytic migration and/or a correlation to the excitotoxicity in neurons in AD [78-80]. Moreover, the APP/PS1dE9 mouse model does not represent the entire AD clinical pathology [32], for example, it does not develop hyperphosphorylated Tau-induced intracellular neurofibrillary Tau tangles. The usage of young double transgenic APP/PS1dE9 mice is a major limitation in our study. We assessed behavior and brain pathology in APP/PS1dE9 mice between the ages of 4 months and 10 months, which would clinically translate to an age of 25-35 human years old with a very early onset familial AD. However, the APP/PS1dE9 mice at 8 months old did not show memory deficits despite of displaying robust hallmark AD brain pathologies.

### Conclusions

Our study aimed to determine the contributions of reactive astrocytes to AD pathogenesis through morphological and functional changes. Using post-mortem human AD brain and transgenic APP/PS1dE9 mouse model brain tissues, we discovered upregulation of NHE1 protein in reactive astrocytes, and this upregulation was significantly increased in proximity to Aβ plaque deposits. Administration of NHE1 inhibitor HOE642 in the APP/PS1dE9 mice starting at 4 months of age attenuated locomotor hyperactivity at 7 months of age. In addition, the early administration of HOE642 also attenuated Aβ peptide fibril load in the 8-month-old APP/PS1dE9 hippocampal and cortical regions. These findings provide evidence for the role of NHE1 protein activity in astrocyte dysfunction and therapeutic potential for targeting NHE1 protein in reducing early AD pathogenesis.

### Supplementary Materials

The Supplementary data can be found online at: www.aginganddisease.org/EN/10.14336/AD.2024.1294.

## References

[1] Khakh BS, Deneen B (2019). The Emerging Nature of Astrocyte Diversity. Annu Rev Neurosci, 42:187-207. 10.1146/annurev-neuro-070918-050443 31283899

[2] Linnerbauer M, Rothhammer V (2020). Protective Functions of Reactive Astrocytes Following Central Nervous System Insult. Front Immunol, 11:573256. 10.3389/fimmu.2020.573256 PMC7561408 33117368

[3] Pekna M, Pekny M (2021). The Complement System: A Powerful Modulator and Effector of Astrocyte Function in the Healthy and Diseased Central Nervous System. Cells, 10. 10.3390/cells10071812 PMC8303424 34359981

[4] Reichenbach N, Delekate A, Plescher M, Schmitt F, Krauss S, Blank N, et al. (2019). Inhibition of Stat3-mediated astrogliosis ameliorates pathology in an Alzheimer's disease model. EMBO Mol Med, 11. 10.15252/emmm.201809665 PMC6365929 30617153

[5] Chun H, Im H, Kang YJ, Kim Y, Shin JH, Won W, et al. (2020). Severe reactive astrocytes precipitate pathological hallmarks of Alzheimer's disease via H(2)O(2)(-) production. Nat Neurosci, 23:1555-1566. 10.1038/s41593-020-00735-y 33199896

[6] Jiwaji Z, Tiwari SS, Avilés-Reyes RX, Hooley M, Hampton D, Torvell M, et al. (2022). Reactive astrocytes acquire neuroprotective as well as deleterious signatures in response to Tau and Aß pathology. Nat Commun, 13:135. 10.1038/s41467-021-27702-w PMC8748982 35013236

[7] Zou K, Abdullah M, Michikawa M (2020). Current Biomarkers for Alzheimer's Disease: From CSF to Blood. J Pers Med, 10. 10.3390/jpm10030085 PMC7564023 32806668

[8] Van Dyck CH, Swanson CJ, Aisen P, Bateman RJ, Chen C, Gee M, et al. (2023). Lecanemab in Early Alzheimer’s Disease. New England Journal of Medicine, 388:9-21. 10.1056/NEJMoa2212948 36449413

[9] Altine-Samey R, Antier D, Mavel S, Dufour-Rainfray D, Balageas AC, Beaufils E, et al. (2021). The contributions of metabolomics in the discovery of new therapeutic targets in Alzheimer's disease. Fundam Clin Pharmacol, 35:582-594. 10.1111/fcp.12654 33484165

[10] Pereira JB, Janelidze S, Smith R, Mattsson-Carlgren N, Palmqvist S, Teunissen CE, et al. (2021). Plasma GFAP is an early marker of amyloid-beta but not tau pathology in Alzheimer's disease. Brain, 144:3505-3516. 10.1093/brain/awab223 PMC8677538 34259835

[11] Johansson C, Thordardottir S, Laffita-Mesa J, Rodriguez-Vieitez E, Zetterberg H, Blennow K, et al. (2023). Plasma biomarker profiles in autosomal dominant Alzheimer's disease. Brain, 146:1132-1140. 10.1093/brain/awac399 PMC9976964 36626935

[12] Kim KY, Shin KY, Chang KA (2023). GFAP as a Potential Biomarker for Alzheimer's Disease: A Systematic Review and Meta-Analysis. Cells, 12. 10.3390/cells12091309 PMC10177296 37174709

[13] Verkhratsky A, Li B, Scuderi C, Parpura V (2021). Principles of Astrogliopathology. Adv Neurobiol, 26:55-73. 10.1007/978-3-030-77375-5_3 PMC8999877 34888830

[14] Ferrer I (2017). Diversity of astroglial responses across human neurodegenerative disorders and brain aging. Brain Pathol, 27:645-674. 10.1111/bpa.12538 PMC8029391 28804999

[15] Assefa BT, Gebre AK, Altaye BM (2018). Reactive Astrocytes as Drug Target in Alzheimer's Disease. Biomed Res Int, 2018:4160247. 10.1155/2018/4160247 PMC5977027 29888263

[16] Begum G, Song S, Wang S, Zhao H, Bhuiyan MIH, Li E, et al. (2018). Selective knockout of astrocytic Na(+)/H(+) exchanger isoform 1 reduces astrogliosis, BBB damage, infarction, and improves neurological function after ischemic stroke. Glia, 66:126-144. 10.1002/glia.23232 PMC5705024 28925083

[17] Liu Q, Bhuiyan MIH, Liu R, Song S, Begum G, Young CB, et al. (2021). Attenuating vascular stenosis-induced astrogliosis preserves white matter integrity and cognitive function. J Neuroinflammation, 18:187. 10.1186/s12974-021-02234-8 PMC8403348 34454529

[18] Wang JL, Xu CJ (2020). Astrocytes autophagy in aging and neurodegenerative disorders. Biomed Pharmacother, 122:109691. 10.1016/j.biopha.2019.109691 31786465

[19] Quick JD, Silva C, Wong JH, Lim KL, Reynolds R, Barron AM, et al. (2023). Lysosomal acidification dysfunction in microglia: an emerging pathogenic mechanism of neuroinflammation and neurodegeneration. J Neuroinflammation, 20:185. 10.1186/s12974-023-02866-y PMC10403868 37543564

[20] Liu R, Wang J, Chen Y, Collier JM, Capuk O, Jin S, et al. (2022). NOX activation in reactive astrocytes regulates astrocytic LCN2 expression and neurodegeneration. Cell Death Dis, 13:371. 10.1038/s41419-022-04831-8 PMC9018876 35440572

[21] Zaqout S, Becker LL, Kaindl AM (2020). Immunofluorescence Staining of Paraffin Sections Step by Step. Front Neuroanat, 14:582218. 10.3389/fnana.2020.582218 PMC7680859 33240048

[22] Percie du Sert N, Hurst V, Ahluwalia A, Alam S, Avey MT, Baker M, et al. (2020). The ARRIVE guidelines 2.0: Updated guidelines for reporting animal research. PLoS Biol, 18:e3000410. 10.1371/journal.pbio.3000410 PMC7360023 32663219

[23] Malm T, Koistinaho J, Kanninen K (2011). Utilization of APPswe/PS1dE9 Transgenic Mice in Research of Alzheimer's Disease: Focus on Gene Therapy and Cell-Based Therapy Applications. Int J Alzheimers Dis, 2011:517160. 10.4061/2011/517160 PMC3205616 22114743

[24] Minkeviciene R, Rheims S, Dobszay MB, Zilberter M, Hartikainen J, Fülöp L, et al. (2009). Amyloid beta-induced neuronal hyperexcitability triggers progressive epilepsy. J Neurosci, 29:3453-3462. 10.1523/JNEUROSCI.5215-08.2009 PMC6665248 19295151

[25] Lee JH, Yang DS, Goulbourne CN, Im E, Stavrides P, Pensalfini A, et al. (2022). Faulty autolysosome acidification in Alzheimer's disease mouse models induces autophagic build-up of Abeta in neurons, yielding senile plaques. Nat Neurosci, 25:688-701. 10.1038/s41593-022-01084-8 PMC9174056 35654956

[26] Wan H, Brathwaite S, Ai J, Hynynen K, Macdonald RL (2021). Role of perivascular and meningeal macrophages in outcome following experimental subarachnoid hemorrhage. J Cereb Blood Flow Metab, 41:1842-1857. 10.1177/0271678X20980296 PMC8327101 33444089

[27] Ritzel RM, Li Y, He J, Khan N, Doran SJ, Faden AI, et al. (2020). Sustained neuronal and microglial alterations are associated with diverse neurobehavioral dysfunction long after experimental brain injury. Neurobiol Dis, 136:104713. 10.1016/j.nbd.2019.104713 PMC7155942 31843705

[28] Chen L, Ma J, Guan Y (2004). Study of an electroosmotic pump for liquid delivery and its application in capillary column liquid chromatography. J Chromatogr A, 1028:219-226. 10.1016/j.chroma.2003.11.071 14989475

[29] Sukoff Rizzo SJ, Anderson LC, Green TL, McGarr T, Wells G, Winter SS (2018). Assessing Healthspan and Lifespan Measures in Aging Mice: Optimization of Testing Protocols, Replicability, and Rater Reliability. Curr Protoc Mouse Biol, 8:e45. 10.1002/cpmo.45 29924918

[30] Escartin C, Galea E, Lakatos A, O'Callaghan JP, Petzold GC, Serrano-Pozo A, et al. (2021). Reactive astrocyte nomenclature, definitions, and future directions. Nat Neurosci, 24:312-325. 10.1038/s41593-020-00783-4 PMC8007081 33589835

[31] Schiweck J, Eickholt BJ, Murk K (2018). Important Shapeshifter: Mechanisms Allowing Astrocytes to Respond to the Changing Nervous System During Development, Injury and Disease. Front Cell Neurosci, 12:261. 10.3389/fncel.2018.00261 PMC6111612 30186118

[32] Jankowsky JL, Fadale DJ, Anderson J, Xu GM, Gonzales V, Jenkins NA, et al. (2004). Mutant presenilins specifically elevate the levels of the 42 residue beta-amyloid peptide in vivo: evidence for augmentation of a 42-specific gamma secretase. Hum Mol Genet, 13:159-170. 10.1093/hmg/ddh019 14645205

[33] Olabarria M, Noristani HN, Verkhratsky A, Rodriguez JJ (2010). Concomitant astroglial atrophy and astrogliosis in a triple transgenic animal model of Alzheimer's disease. Glia, 58:831-838. 10.1002/glia.20967 20140958

[34] Kayed R, Head E, Sarsoza F, Saing T, Cotman CW, Necula M, et al. (2007). Fibril specific, conformation dependent antibodies recognize a generic epitope common to amyloid fibrils and fibrillar oligomers that is absent in prefibrillar oligomers. Mol Neurodegener, 2:18. 10.1186/1750-1326-2-18 PMC2100048 17897471

[35] Wang T, Chen Y, Zou Y, Pang Y, He X, Chen Y, et al. (2022). Locomotor Hyperactivity in the Early-Stage Alzheimer's Disease-like Pathology of APP/PS1 Mice: Associated with Impaired Polarization of Astrocyte Aquaporin 4. Aging Dis, 13:1504-1522. 10.14336/AD.2022.0219 PMC9466968 36186142

[36] Keszycki RM, Fisher DW, Dong H (2019). The Hyperactivity-Impulsivity-Irritiability-Disinhibition-Aggression-Agitation Domain in Alzheimer's Disease: Current Management and Future Directions. Front Pharmacol, 10:1109. 10.3389/fphar.2019.01109 PMC6777414 31611794

[37] Paumier A, Boisseau S, Jacquier-Sarlin M, Pernet-Gallay K, Buisson A, Albrieux M (2022). Astrocyte-neuron interplay is critical for Alzheimer's disease pathogenesis and is rescued by TRPA1 channel blockade. Brain, 145:388-405. 10.1093/brain/awab281 34302466

[38] Upadhyay A, Chhangani D, Rao NR, Kofler J, Vassar R, Rincon-Limas DE, et al. (2023). Amyloid fibril proteomics of AD brains reveals modifiers of aggregation and toxicity. Mol Neurodegener, 18:61. 10.1186/s13024-023-00654-z PMC10503190 37710351

[39] Verkhratsky A, Rodrigues JJ, Pivoriunas A, Zorec R, Semyanov A (2019). Astroglial atrophy in Alzheimer's disease. Pflugers Arch, 471:1247-1261. 10.1007/s00424-019-02310-2 31520182

[40] Ebenau JL, Pelkmans W, Verberk IMW, Verfaillie SCJ, van den Bosch KA, van Leeuwenstijn M, et al. (2022). Association of CSF, Plasma, and Imaging Markers of Neurodegeneration With Clinical Progression in People With Subjective Cognitive Decline. Neurology, 98:e1315-e1326. 10.1212/WNL.0000000000200035 PMC8967429 35110378

[41] Verkhratsky A, Zorec R, Parpura V (2017). Stratification of astrocytes in healthy and diseased brain. Brain Pathol, 27:629-644. 10.1111/bpa.12537 PMC5599174 28805002

[42] Endo F, Kasai A, Soto JS, Yu X, Qu Z, Hashimoto H, et al. (2022). Molecular basis of astrocyte diversity and morphology across the CNS in health and disease. Science, 378:eadc9020. 10.1126/science.adc9020 PMC9873482 36378959

[43] Sadick JS, O'Dea MR, Hasel P, Dykstra T, Faustin A, Liddelow SA (2022). Astrocytes and oligodendrocytes undergo subtype-specific transcriptional changes in Alzheimer's disease. Neuron, 110:1788-1805 e1710. 10.1016/j.neuron.2022.03.008 PMC9167747 35381189

[44] Hasel P, Rose IVL, Sadick JS, Kim RD, Liddelow SA (2021). Neuroinflammatory astrocyte subtypes in the mouse brain. Nat Neurosci, 24:1475-1487. 10.1038/s41593-021-00905-6 34413515

[45] Frost GR, Li YM (2017). The role of astrocytes in amyloid production and Alzheimer's disease. Open Biol, 7. 10.1098/rsob.170228 PMC5746550 29237809

[46] Wang P, Li L, Zhang Z, Kan Q, Gao F, Chen S (2016). Time-dependent activity of Na+/H+ exchanger isoform 1 and homeostasis of intracellular pH in astrocytes exposed to CoCl2 treatment. Mol Med Rep, 13:4443-4450. 10.3892/mmr.2016.5067 27035646

[47] Onos KD, Uyar A, Keezer KJ, Jackson HM, Preuss C, Acklin CJ, et al. (2019). Enhancing face validity of mouse models of Alzheimer's disease with natural genetic variation. PLoS Genet, 15:e1008155. 10.1371/journal.pgen.1008155 PMC6576791 31150388

[48] Perea G, Navarrete M, Araque A (2009). Tripartite synapses: astrocytes process and control synaptic information. Trends Neurosci, 32:421-431. 10.1016/j.tins.2009.05.001 19615761

[49] Shah D, Gsell W, Wahis J, Luckett ES, Jamoulle T, Vermaercke B, et al. (2022). Astrocyte calcium dysfunction causes early network hyperactivity in Alzheimer's disease. Cell Rep, 40:111280. 10.1016/j.celrep.2022.111280 PMC9433881 36001964

[50] Song S, Luo L, Sun B, Sun D (2020). Roles of glial ion transporters in brain diseases. Glia, 68:472-494. 10.1002/glia.23699 PMC6957693 31418931

[51] Yamamoto T, Shirayama T, Takahashi T, Matsubara H (2009). Altered expression of Na+ transporters at the mRNA level in rat normal and hypertrophic myocardium. Heart Vessels, 24:54-62. 10.1007/s00380-008-1071-8 19165570

[52] Zhao X, Gorin FA, Berman RF, Lyeth BG (2008). Differential hippocampal protection when blocking intracellular sodium and calcium entry during traumatic brain injury in rats. J Neurotrauma, 25:1195-1205. 10.1089/neu.2008.0635 PMC2652584 18847376

[53] Lo CH, Zeng J (2023). Defective lysosomal acidification: a new prognostic marker and therapeutic target for neurodegenerative diseases. Transl Neurodegener, 12:29. 10.1186/s40035-023-00362-0 PMC10249214 37287072

[54] Gonzales EB, Sumien N (2017). Acidity and Acid-Sensing Ion Channels in the Normal and Alzheimer's Disease Brain. J Alzheimers Dis, 57:1137-1144. 10.3233/JAD-161131 28211811

[55] Coffey EE, Beckel JM, Laties AM, Mitchell CH (2014). Lysosomal alkalization and dysfunction in human fibroblasts with the Alzheimer's disease-linked presenilin 1 A246E mutation can be reversed with cAMP. Neuroscience, 263:111-124. 10.1016/j.neuroscience.2014.01.001 PMC4028113 24418614

[56] de Leeuw SM, Kirschner AWT, Lindner K, Rust R, Budny V, Wolski WE, et al. (2022). APOE2, E3, and E4 differentially modulate cellular homeostasis, cholesterol metabolism, and inflammatory response in isogenic iPSC-derived astrocytes. Stem Cell Reports, 17:110-126. 10.1016/j.stemcr.2021.11.007 PMC8758949 34919811

[57] Ledo JH, Liebmann T, Zhang R, Chang JC, Azevedo EP, Wong E, et al. (2021). Presenilin 1 phosphorylation regulates amyloid-beta degradation by microglia. Mol Psychiatry, 26:5620-5635. 10.1038/s41380-020-0856-8 PMC7881060 32792660

[58] Lee H, Cho S, Kim MJ, Park YJ, Cho E, Jo YS, et al. (2023). ApoE4-dependent lysosomal cholesterol accumulation impairs mitochondrial homeostasis and oxidative phosphorylation in human astrocytes. Cell Rep, 42:113183. 10.1016/j.celrep.2023.113183 37777962

[59] Prasad H, Rao R (2018). Amyloid clearance defect in ApoE4 astrocytes is reversed by epigenetic correction of endosomal pH. Proc Natl Acad Sci U S A, 115:E6640-E6649. 10.1073/pnas.1801612115 PMC6048470 29946028

[60] Sivanesan S, Mundugaru R, Rajadas J (2018). Possible Clues for Brain Energy Translation via Endolysosomal Trafficking of APP-CTFs in Alzheimer's Disease. Oxid Med Cell Longev, 2018:2764831. 10.1155/2018/2764831 PMC6215552 30420907

[61] Song S, Yu L, Hasan MN, Paruchuri SS, Mullett SJ, Sullivan MLG, et al. (2022). Elevated microglial oxidative phosphorylation and phagocytosis stimulate post-stroke brain remodeling and cognitive function recovery in mice. Commun Biol, 5:35. 10.1038/s42003-021-02984-4 PMC8752825 35017668

[62] Hasan MN, Luo L, Ding D, Song S, Bhuiyan MIH, Liu R, et al. (2021). Blocking NHE1 stimulates glioma tumor immunity by restoring OXPHOS function of myeloid cells. Theranostics, 11:1295-1309. 10.7150/thno.50150 PMC7738877 33391535

[63] Giannaki M, Schrodl-Haussel M, Khakipoor S, Kirsch M, Roussa E (2021). STAT3-dependent regulation of the electrogenic Na(+/) HCO(3)(-) cotransporter 1 (NBCe1) functional expression in cortical astrocytes. J Cell Physiol, 236:2036-2050. 10.1002/jcp.29990 32761631

[64] Lee Y, Miller MR, Fernandez MA, Berg EL, Prada AM, Ouyang Q, et al. (2022). Early lysosome defects precede neurodegeneration with amyloid-beta and tau aggregation in NHE6-null rat brain. Brain, 145:3187-3202. 10.1093/brain/awab467 PMC10147331 34928329

[65] Karmazyn M, Pierce GN, Fliegel L (2022). The Remaining Conundrum of the Role of the Na(+)/H(+) Exchanger Isoform 1 (NHE1) in Cardiac Physiology and Pathology: Can It Be Rectified? Rev Cardiovasc Med, 23:284. 10.31083/j.rcm2308284 PMC11266974 39076631

[66] Karmazyn M (2013). NHE-1: still a viable therapeutic target. J Mol Cell Cardiol, 61:77-82. 10.1016/j.yjmcc.2013.02.006 23429008

[67] Kawamoto T, Kimura H, Kusumoto K, Fukumoto S, Shiraishi M, Watanabe T, et al. (2001). Potent and selective inhibition of the human Na+/H+ exchanger isoform NHE1 by a novel aminoguanidine derivative T-162559. Eur J Pharmacol, 420:1-8. 10.1016/s0014-2999(01)00991-8 11412833

[68] Verma V, Bali A, Singh N, Jaggi AS (2015). Implications of sodium hydrogen exchangers in various brain diseases. J Basic Clin Physiol Pharmacol, 26:417-426. 10.1515/jbcpp-2014-0117 26020555

[69] Ma E, Haddad GG (1997). Expression and localization of Na+/H+ exchangers in rat central nervous system. Neuroscience, 79:591-603. 10.1016/s0306-4522(96)00674-4 9200742

[70] Orlowski J, Kandasamy RA, Shull GE (1992). Molecular cloning of putative members of the Na/H exchanger gene family. cDNA cloning, deduced amino acid sequence, and mRNA tissue expression of the rat Na/H exchanger NHE-1 and two structurally related proteins. J Biol Chem, 267:9331-9339. 1577762

[71] Harguindey S, Arranz JL, Polo Orozco JD, Rauch C, Fais S, Cardone RA, et al. (2013). Cariporide and other new and powerful NHE1 inhibitors as potentially selective anticancer drugs--an integral molecular/biochemical/metabolic/clinical approach after one hundred years of cancer research. J Transl Med, 11:282. 10.1186/1479-5876-11-282 PMC3826530 24195657

[72] Donowitz M, Ming Tse C, Fuster D (2013). SLC9/NHE gene family, a plasma membrane and organellar family of Na(+)/H(+) exchangers. Mol Aspects Med, 34:236-251. 10.1016/j.mam.2012.05.001 PMC3724465 23506868

[73] Chaitman BR (2003). A review of the GUARDIAN trial results: clinical implications and the significance of elevated perioperative CK-MB on 6-month survival. J Card Surg, 18 Suppl 1:13-20. 10.1046/j.1540-8191.18.s1.3.x 12691375

[74] Mentzer RM Jr., Bartels C, Bolli R, Boyce S, Buckberg GD, Chaitman B, et al. (2008). Sodium-hydrogen exchange inhibition by cariporide to reduce the risk of ischemic cardiac events in patients undergoing coronary artery bypass grafting: results of the EXPEDITION study. Ann Thorac Surg, 85:1261-1270. 10.1016/j.athoracsur.2007.10.054 18355507

[75] Chang HB, Gao X, Nepomuceno R, Hu S, Sun D (2015). Na(+)/H(+) exchanger in the regulation of platelet activation and paradoxical effects of cariporide. Exp Neurol, 272:11-16. 10.1016/j.expneurol.2014.12.023 PMC4500746 25595121

[76] Ghaleh B, Barthelemy I, Wojcik J, Sambin L, Bize A, Hittinger L, et al. (2020). Protective effects of rimeporide on left ventricular function in golden retriever muscular dystrophy dogs. Int J Cardiol, 312:89-95. 10.1016/j.ijcard.2020.03.031 32199683

[77] Adam SA, Schnell O, Poschl J, Eigenbrod S, Kretzschmar HA, Tonn JC, et al. (2012). ALDH1A1 is a marker of astrocytic differentiation during brain development and correlates with better survival in glioblastoma patients. Brain Pathol, 22:788-797. 10.1111/j.1750-3639.2012.00592.x PMC8057636 22417385

[78] Yeung JHY, Palpagama TH, Wood OWG, Turner C, Waldvogel HJ, Faull RLM, et al. (2021). EAAT2 Expression in the Hippocampus, Subiculum, Entorhinal Cortex and Superior Temporal Gyrus in Alzheimer's Disease. Front Cell Neurosci, 15:702824. 10.3389/fncel.2021.702824 PMC8475191 34588956

[79] Zhang Z, Ma Z, Zou W, Guo H, Liu M, Ma Y, et al. (2019). The Appropriate Marker for Astrocytes: Comparing the Distribution and Expression of Three Astrocytic Markers in Different Mouse Cerebral Regions. Biomed Res Int, 2019:9605265. 10.1155/2019/9605265 PMC6613026 31341912

[80] Brozzi F, Arcuri C, Giambanco I, Donato R (2009). S100B Protein Regulates Astrocyte Shape and Migration via Interaction with Src Kinase: IMPLICATIONS FOR ASTROCYTE DEvelopment, activation, and tumor growth. J Biol Chem, 284:8797-8811. 10.1074/jbc.M805897200 PMC2659238 19147496

