# Supplementary material for: Upregulation of Na/H Exchanger in Astrogliosis and Early Alzheimer’s Disease Pathogenesis
Source: Aging Dis. 2024 Dec 15;16(6):3546–66. doi: 10.14336/AD.2024.1294 (PMC12539526; doi:10.14336/AD.2024.1294)
Supplement: Supplementary file 1 — The Supplementary data can be found online at: www.aginganddisease.org/EN/10.14336/AD.2024.1294. [file AD-16-6-3546-s.pdf]

SUPPLEMENTARY DATA

Upregulation of Na/H Exchanger in Astrogliosis and Early Alzheimer’s Disease Pathogenesis

Jenelle M. Collier, Shamseldin Metwally, Mary McFarland, Sanjana Krishna, Pallavi Kurella, Victoria Fiesler, Mark Stauffer, Gulnaz Begum, Julia Kofler, Dandan Sun

© 2024. Collier JM. et al. Published online at http://www.aginganddisease.org/EN/10.14336/AD.2024.1294

SUPPLEMENTARY DATA

Supplemental Table 1: List of Antibodies used for this study

Primary Antibody	Dilution	Catalog #	Vendor
**GFAP, rabbit**	**1:500**	**Z0334**	**DAKO**
**GFAP, chicken**	**1:100**	**ab4674**	**Abcam**
**NHE1, mouse**	**1:100**	**Sc-136239**	**Santa Cruz**
**APP/β-Amyloid (NAB228), mouse**	**1:1000**	**2450**	**Cell Signaling**
**Amyloid Fibril (OC), rabbit**	**1:200**	**200-401-E87**	**Rockland**
**Secondary Antibody/Dyes**	**Dilution**	**Catalog #**	**Vendor**
**Alexa 488, mouse**	**1:200**	**A11029**	**Invitrogen**
**Alexa 488, chicken**	**1:200**	**A11039**	**Invitrogen**
**Alexa 546, rabbit**	**1:200**	**A10035**	**Invitrogen**
**Alexa Fluor 488 AffiniPure Fab Fragment Donkey, mouse**	**1:200**	**715-547-003**	**Jackson Immuno Research Laboratories**
**Thioflavin S**	**1:1000**	**T1892-25G**	**Sigma-Aldrich**
**DAPI**	**1:1000**	**D1306**	**Invitrogen**
**TO-PRO-3**	**1:1000**	**R37113**	**Invitrogen**

Primary and Secondary Antibodies and Nuclear and Aβ dyes used throughout the entirety of this study are listed with species reactivity, dilutions, catalog numbers, and vendors.

© 2024. Collier JM. et al. Published online at http://www.aginganddisease.org/EN/10.14336/AD.2024.1294

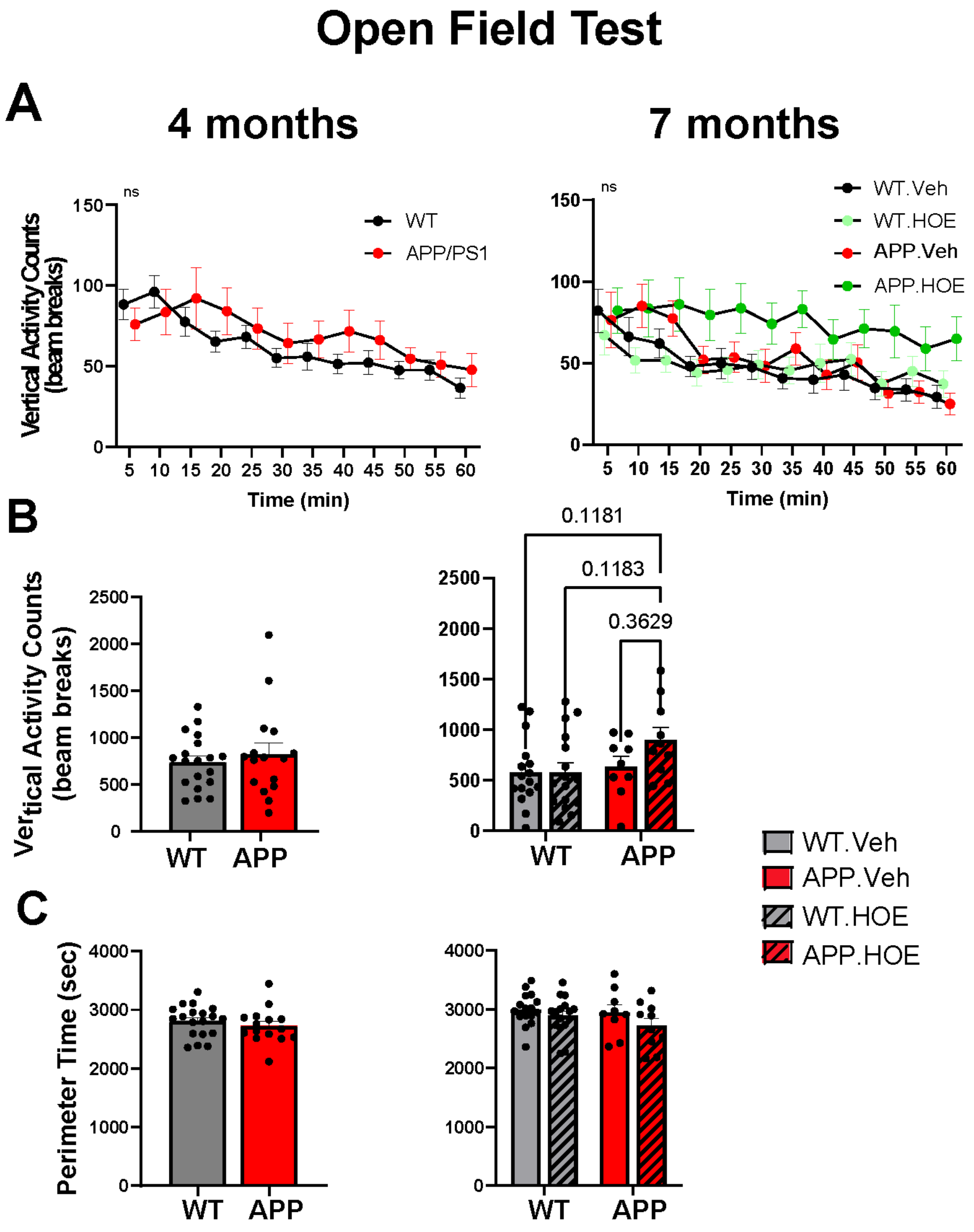

Supplementary Figure 1. Open Field Test Behavior A. Open Field Test in 4-month old of WT and APP, and 7-month old of WT.Veh, WT.HOE, APP.Veh, and APP.HOE mice. Lined graphs display vertical activity differences between the groups. No significant differences were detected between the groups at 4-month and 7-month old. **B.** Total Vertical Activity Counts show no significant differences between either the genotype or treatment groups. **C.** Total Perimeter Time reveals no significant differences between the genotype or treatment groups.

© 2024. Collier JM. et al. Published online at http://www.aginganddisease.org/EN/10.14336/AD.2024.1294

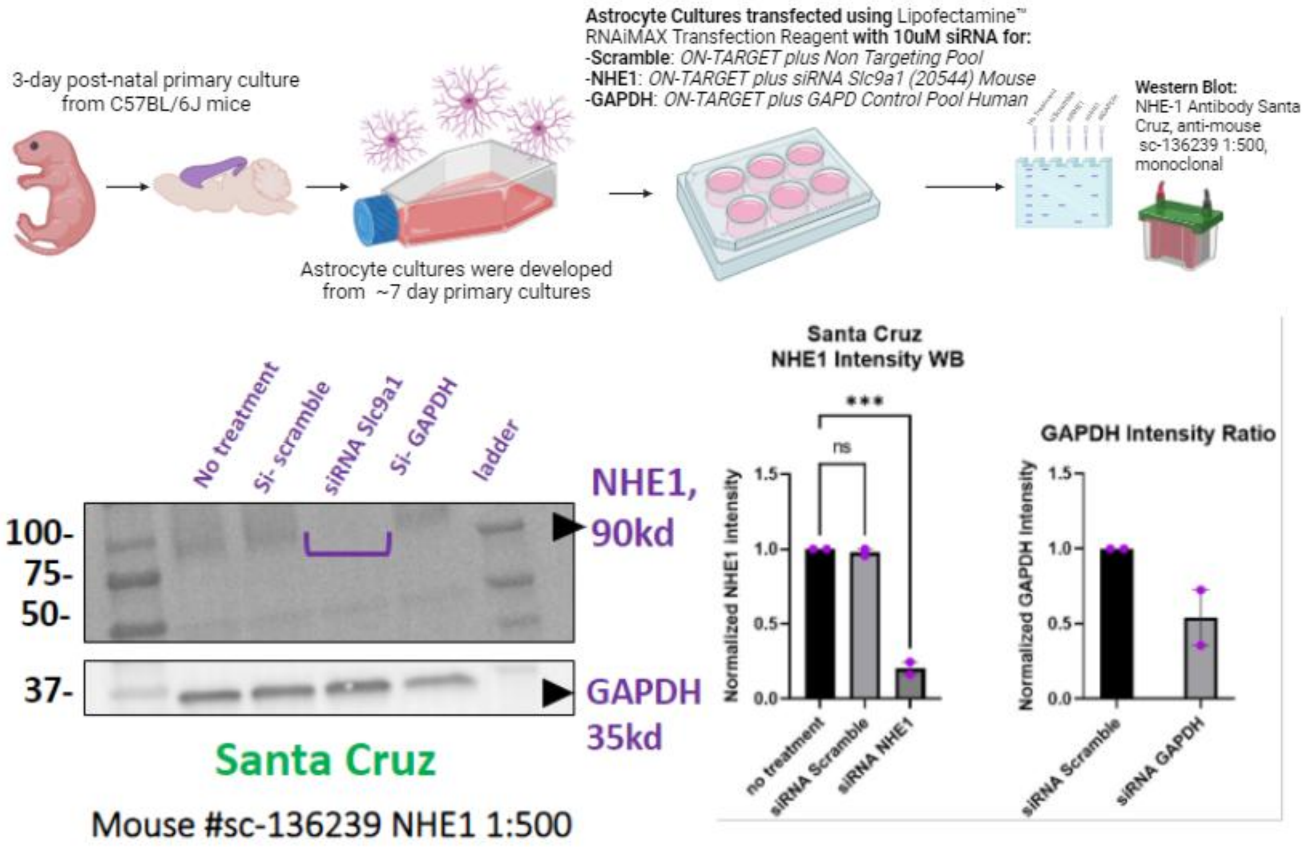

Supplementary Figure 2. Validation of Santa Cruz anti-NHE1 antibody in astrocyte cultures with targeting Slc9a1 siRNA. A. P3 mouse pup cortical tissues were isolated and used for astrocyte cultures. Cultures were transfected with Lipofectamine RNAiMAX transfection Reagent with 10µM of either non-targeting pool scramble siRNAs, Slc9a1 siRNAs, or GAPDH siRNAs for 3 days. **B.** NHE1 protein in mouse primary astrocytes was quantified with Western Blotting probed with Anti-mouse NHE1 antibody (Santa Cruz #sc-136239). 80% of NHE1 protein was reduced in cells treated with Slc9a1 (L-048336-01-0005) siRNA (***$p<0.001$) but not by scramble or GAPDH (D-0011830-10-05) siRNAs. Data are mean ±SEM of 3 (independent experiments). One-way ANOVA with Fisher's LSD Multiple Comparison test (for unequal replications).

© 2024. Collier JM. et al. Published online at http://www.aginganddisease.org/EN/10.14336/AD.2024.1294

SUPPLEMENTARY DATA

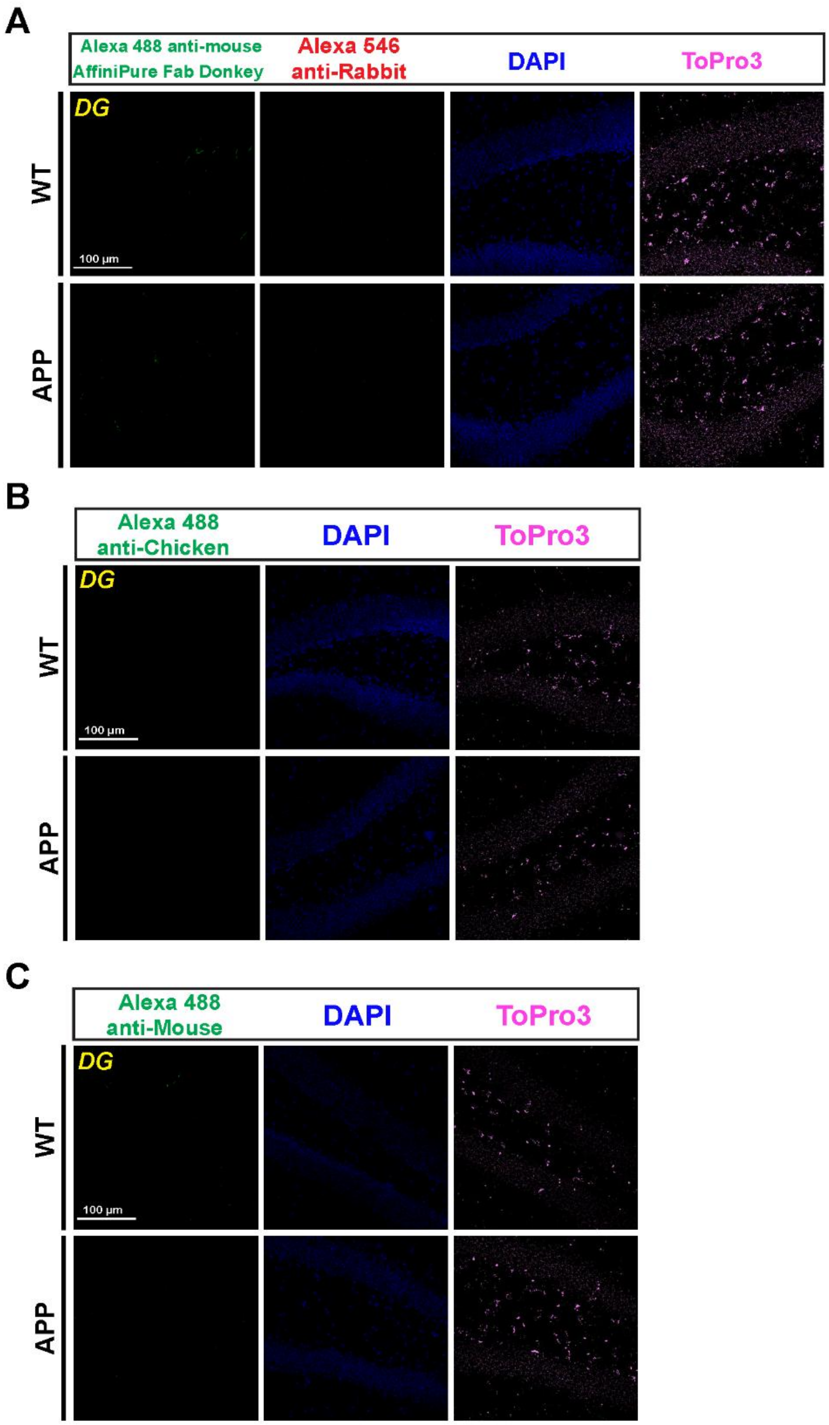

Supplementary Figure 3. Immunostaining negative controls. A. Negative control staining of 8-month WT and APP mice brains. Confocal images of hippocampal dentate gyrus stained with secondary antibody anti-Mouse Alexa Fluor 488 Affini Pure Fab Fragment Donkey or anti-rabbit Alexa 546 along with DAPI and ToPro3 nuclear dyes. **B.** Confocal images of secondary antibody staining with anti-Chicken Alexa 488. **C.** Confocal images of secondary antibody staining with anti-mouse Alexa 488.

© 2024. Collier JM. et al. Published online at http://www.aginganddisease.org/EN/10.14336/AD.2024.1294
